# Establishment of the TALE-code reveals aberrantly activated homeobox gene PBX1 in Hodgkin lymphoma

**DOI:** 10.1371/journal.pone.0246603

**Published:** 2021-02-04

**Authors:** Stefan Nagel, Claudia Pommerenke, Corinna Meyer, Roderick A. F. MacLeod, Hans G. Drexler

**Affiliations:** Department of Human and Animal Cell Lines, Leibniz-Institute DSMZ – German Collection of Microorganisms and Cell Cultures, Braunschweig, Germany; Beth Israel Deaconess Medical Center-Harvard Medical School, UNITED STATES

## Abstract

Homeobox genes encode transcription factors which regulate basic processes in development and cell differentiation and are grouped into classes and subclasses according to sequence similarities. Here, we analyzed the activities of the 20 members strong TALE homeobox gene class in early hematopoiesis and in lymphopoiesis including developing and mature B-cells, T-cells, natural killer (NK)-cells and innate lymphoid cells (ILC). The resultant expression pattern comprised eleven genes and which we termed TALE-code enables discrimination of normal and aberrant activities of TALE homeobox genes in lymphoid malignancies. Subsequent expression analysis of TALE homeobox genes in public datasets of Hodgkin lymphoma (HL) patients revealed overexpression of IRX3, IRX4, MEIS1, MEIS3, PBX1, PBX4 and TGIF1. As paradigm we focused on PBX1 which was deregulated in about 17% HL patients. Normal PBX1 expression was restricted to hematopoietic stem cells and progenitors of T-cells and ILCs but absent in B-cells, reflecting its roles in stemness and early differentiation. HL cell line SUP-HD1 expressed enhanced PBX1 levels and served as an in vitro model to identify upstream regulators and downstream targets in this malignancy. Genomic studies of this cell line therein showed a gain of the PBX1 locus at 1q23 which may underlie its aberrant expression. Comparative expression profiling analyses of HL patients and cell lines followed by knockdown experiments revealed NFIB and TLX2 as target genes activated by PBX1. HOX proteins operate as cofactors of PBX1. Accordingly, our data showed that HOXB9 overexpressed in HL coactivated TLX2 but not NFIB while activating TNFRSF9 without PBX1. Further downstream analyses showed that TLX2 activated TBX15 which operated anti-apoptotically. Taken together, we discovered a lymphoid TALE-code and identified an aberrant network around deregulated TALE homeobox gene PBX1 which may disturb B-cell differentiation in HL by reactivation of progenitor-specific genes. These findings may provide the framework for future studies to exploit possible vulnerabilities of malignant cells in therapeutic scenarios.

## Introduction

To produce the complete panel of blood and immune cells hematopoiesis begins with hematopoietic stem cells (HSCs) in the bone marrow. HSCs and lymphomyeloid-primed progenitors (LMPP) generate common myeloid progenitors (CMP) and common lymphoid progenitors (CLP). CMPs initiate the differentiation into all myeloid cells while CLPs produce all types of lymphocytes comprising B-cells, T-cells, natural killer (NK)-cells and innate lymphoid cells (ILC). The development of B-cells begins with the CLP-derived B-cell progenitor (BCP), which in turn differentiates via pro-B-cells into pre-B-cells. The final differentiation steps into memory B-cells and plasma cells via naïve and germinal center (GC) B-cells occur outside of the bone marrow in lymph nodes and spleen [1].

Lymphopoiesis including B-cell development is regulated mainly at the transcriptional level [2,3]. Accordingly, several transcription factors (TFs) like BCL6, EBF1, NKX6-3 and PAX5 generate a B-cell specific regulatory network controlling basic differentiation processes [4–6]. Deregulation of these TFs by chromosomal rearrangement, gene mutation or Epstein-Barr virus infection underlies the generation of B-cell malignancies [7–9]. Thus, the analysis of particular developmental TFs may illuminate our understanding of both normal lymphopoiesis and lymphoid neoplasms.

Homeobox genes encode TFs containing a 60 amino acid residue homeodomain which allows both sequence-specific DNA-binding and contacts to cofactors. This domain consists of three alpha-helices which are separated by two loops. The third helix interacts with the major groove of the DNA molecule and confers contact specificity [10]. Most of these TFs regulate basic developmental processes in both embryos and adults, including organization of embryonic body parts, operation as master factor for particular organs, or differentiation of cell lineages and cell types [11–14]. Homeobox genes fall into classes and subclasses according to sequence similarities in their conserved homeobox. The scheme generated comprises eleven classes including the TALE and ANTP classes the latter of which contains the HOXL and NKL subclasses [15].

The clustered HOX genes belong to the HOXL subclass and are expressed within the embryo in a particular pattern termed HOX-code. This code determines the anterior-posterior differentiation of the branchial and head region [11]. HOX proteins cooperate with homeodomain proteins of the TALE class. These proteins possess additional three amino acid residues in the first loop of their conserved homeodomain. TALE, accordingly, stands for Three Amino acids Loop Extension. TALE homeobox genes represent a very ancient group, indicating essential regulatory functions [16]. The human genome contains 20 members of the TALE homeobox gene class [17].

Throughout the recent years, we have created the NKL-code which describes normal expression patterns of NKL homeobox subclass genes in the course of blood cell development covering early hematopoiesis, myelopoiesis and lymphopoiesis [6,18–20]. Gene codes like the HOX- or TLX-code are generated by closely related homeobox genes showing similarities in their encoded homeodomains and flanking regions. They perform similar functions and operate in particular tissue compartments. Here, we followed the same approach of expression analysis used for the generation of the lymphoid NKL-code for the examination of TALE homeobox genes in early hematopoiesis and in lymphopoiesis. We termed the resultant expression pattern TALE-code which allowed identification of deregulated TALE homeobox genes in lymphoid malignancies including Hodgkin lymphoma (HL).

Hodgkin and Reed-Sternberg (HRS) cells, the assumed malignant cells in HL, derive from developing B-cells and typically show abnormalities in B-cell differentiation, apoptosis, and cell communication [21]. Several TFs involved in B-cell development are downregulated which results in incomplete cell differentiation [22–24]. Inhibition of apoptosis and aberrantly activated NFkB-signalling represent additional hallmarks of HL [25]. Finally, HL cells express various autoregulatory interleukins and receptors which attract or deceive immune cells in their vicinity [21]. Because the pathogenesis of HL is so intricate, identifying the players involved and their vulnerabilities may help to develop a framework for novel therapeutic strategies. Here, we exploited the TALE-code established in this study to reveal an aberrant gene regulatory network around PBX1 implicated in deregulation of B-cell differentiation in HL.

## Materials and methods

### Expression profiling and RNA-seq data analysis

Public gene expression profiling datasets used in this study were generated by U133 Plus 2.0 gene chips from Affymetrix and obtained from Gene Expression Omnibus (GEO, www.ncbi.nlm.nih.gov). In addition, GEO provides the online tool GEOR which allows bioinformatic comparison of two defined sample groups. For our screening approach we exploited both expression profiling data and RNA-sequencing data as described previously [6,18,20]. Dataset GSE56315 was used for analysis of developing B-cells [26], and dataset GSE72642 for mature lymphocytes including B-cells, CD4-positive T-cells, CD8-positive T-cells, and NK-cells [27]. Dataset GSE69239 provides RNA-seq data of several isolated hematopoietic entities including HSC, LMPP, CLP, BCP, and different stages of developing T-cells [28]. The following samples from this dataset have been renamed as Thy1 (CD34+ CD7- CD1a-) into DN1, Thy2 (CD34+ CD7+ CD1a-) into DN2, Thy3 (CD34+ CD7+ CD1a+) into DN3, Thy4 (CD4+ CD8+) into DP, Thy5 (CD4+ CD8-) into SP4, and Thy6 (CD4- CD8+) into SP8. Expression data are in units of FPKM (fragments per kilobase of mappable gene length and million reads). RNA-seq data of ILCs and ILC progenitors (ILCP) were obtained from datasets GSE112591 and GSE90834, respectively [29,30]. The expression profiling datasets GSE12453 and GSE39134 were used for analysis of HL patient samples [31,32].

Gene expression profiling data for HL cell lines were generated as we published previously (GSE115191) [33]. Corresponding data for SUP-HD1 and HDLM-2 cells treated for siRNA-mediated knockdown of NFIB and HOXB9, respectively, were generated at the Genome Analytics Facility, Helmholtz Centre for Infection Research (HZI, Braunschweig, Germany). For analysis primary data were transformed as follows: after RMA-background correction and quantile normalization of the spot intensities, profiling data were expressed as ratios of the sample mean and subsequently log2 transformed. Data processing was performed via R/Bioconductor using Limma and Affy packages. Cell lines used to generate RNA-seq data from 100 hematopoietic cell lines including HL are deposited at the cell lines bank DSMZ. Data are available at ArrayExpress via E-MTAB-7721 [34]. Corresponding gene expression values were visualized using shinyNGS (https://github.com/pinin4fjords/shinyngs). Visualization and analysis of the sequences to evaluate splicing forms was performed using the Integrative Genomics Viewer (www.software.broadinstitute.org).

### Cell lines and treatments

Cell lines were obtained from the DSMZ (Braunschweig, Germany) and cultivated as described elsewhere [35]. All cell lines have been authenticated and were tested negative for mycoplasma infection. To modify gene expression levels we used gene specific siRNA oligonucleotides with reference to AllStars negative Control siRNA (siCTR) obtained from Qiagen (Hilden, Germany). SiRNAs (80 pmol) were transfected into 1x10^6^ cells by electroporation using the EPI-2500 impulse generator (Fischer, Heidelberg, Germany) at 350 V for 10 ms. Electroporated cells were harvested after 20 h cultivation. Cells were treated for 16 h with 20 ng/ml recombinant HGF, BMP4 or TGFb (R & D Systems, Abingdon, UK), with MET-receptor inhibitor tivantinib (Calbiochem, Darmstadt, Germany), BMP-receptor inhibitor dorsomorphin (Sigma, Taufkirchen, Germany), or apoptosis-inducer etoposide (Sigma) dissolved in dimethyl sulfoxide (DMSO) at final concentrations of 5 μM, 10 μM or 100μM, respectively.

Apoptosis was analyzed using the IncuCyte S3 Live-Cell Analysis System (Essen Bioscience, Hertfordshire, UK). For detection of apoptotic cells we used the IncuCyte Caspase-3/7 Green Apoptosis Assay diluted at 1:2000 (Essen Bioscience). Live-cell imaging experiments were performed twice with fourfold parallel tests.

### Polymerase chain-reaction (PCR) analyses

Total RNA was extracted from cultivated cell lines using TRIzol reagent (Invitrogen, Darmstadt, Germany). Primary human total RNA derived from bone marrow, intestine, adrenal gland and kidney was purchased from Biochain/BioCat (Heidelberg, Germany), and RNA from peripheral CD19-positive B-cells and CD34-positive HSCs from Miltenyi Biotec (Bergisch Gladbach, Germany). cDNA was synthesized using 5 μg RNA, random priming and Superscript II (Invitrogen). Real time quantitative (RQ)-PCR analysis was performed using the 7500 Real-time System and commercial buffer and primer sets (Applied Biosystems/Life Technologies, Darmstadt, Germany). For normalization of expression levels we quantified the transcripts of TATA box binding protein (TBP).

For copy number quantification we extracted genomic DNA using the High Pure PCR Template Preparation Kit (Roche Diagnostics, Mannheim, Germany). Quantification of PBX1 gene copy numbers was performed with reference to the MEF2C control. The following oligonucleotides (obtained from Eurofins MWG, Ebersberg, Germany) were used: PBX1-for 5`-TAAGGAGGTTGGCAGGATGCTAC-3´, PBX1-rev 5´-GTCAGCCGGGATGCGATTGCTGG-3´, MEF2C-for 5´-GCAGGAATTTGGGAACTGAG-3´, MEF2C-rev 5´-CCCATAGTCCCCGTTTTTCT-3´.

Quantitative analyses were performed twice and measured in triplicate. Standard deviations are presented in the figures as error bars. Statistical significance was assessed by Student´s T-Test (two-tailed) and the calculated p-values were indicated by asterisks (* p<0.05, ** p<0.01, *** p<0.001, n.s. not significant).

For detection of TCF3-PBX1 fusion transcript (also known as E2A-PBX1) and ETV6 control, we performed reverse transcription (RT)-PCR, using previously described oligonucleotides E2A-C and PBX-D purchased from Eurofins MWG [36]. The B-cell line 697 served as positive control. PCR products were generated using taqpol (Qiagen) and thermocycler TGradient (Biometra, Göttingen, Germany), analyzed by gel electrophoresis, and documented with the Azure c200 Gel Imaging System (Azure Biosystems, Dublin, CA, USA).

### Protein analysis

Western blots were generated by the semi-dry method. Protein lysates from cell lines were prepared using SIGMAFast protease inhibitor cocktail (Sigma). Proteins were transferred onto nitrocellulose membranes (Bio-Rad, München, Germany) and blocked with 5% dry milk powder dissolved in phosphate-buffered-saline buffer (PBS). The following antibodies were used: alpha-Tubulin (Sigma, #T6199), PBX1 (LifeSpan Biosciences, Eching, Germany, #LS-C133363), and NFIB (Novus Biologicals, Abingdon, UK, #NBP1-81000). For loading control blots were reversibly stained with Poinceau (Sigma) and detection of alpha-Tubulin (TUBA) performed thereafter. Secondary antibodies were linked to peroxidase for detection by Western-Lightning-ECL (Perkin Elmer, Waltham, MA, USA). Documentation was performed using the digital system ChemoStar Imager (INTAS, Göttingen, Germany).

### Chromosomal and genomic analyses

Karyotyping was performed as described previously [37]. For genomic profiling genomic DNA of HL cell lines was prepared by the Qiagen Gentra Puregene Kit (Qiagen). Labelling, hybridization and scanning of Cytoscan HD arrays was performed at the Genome Analytics Facility (HZI), according to the manufacturer´s protocols (Affymetrix, High Wycombe, UK). Data were interpreted using the Chromosome Analysis Suite software version 3.1.0.15 (Affymetrix). Genomic profiling data were used to determine copy number alterations.

## Results

### Normal TALE homeobox gene expression in lymphopoiesis

**Fig 1:**
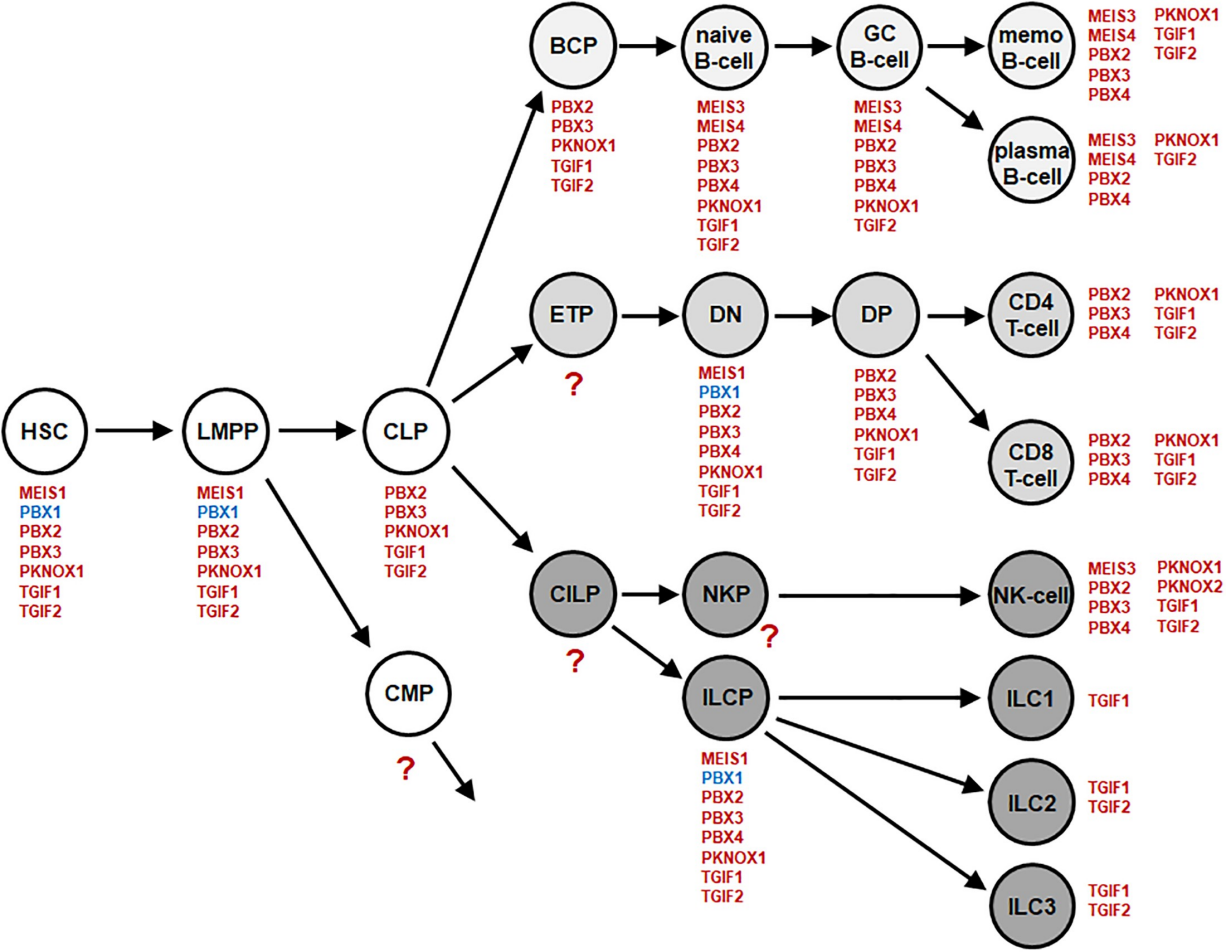
Lymphoid TALE-code. This diagram summarizes the screening results for expression of TALE homeobox genes (red) in early hematopoiesis and lymphopoiesis. We have termed this expression pattern TALE-code. Expression of PBX1 (blue) was detected in HSC, LMPP, DN T-cells and ILCP. BCP: B-cell progenitor, CILP: common innate lymphoid progenitor, CLP: common lymphoid progenitor, CMP: common myeloid progenitor, DN: double negative T-cell, DP: double positive T-cell, ETP: early T-cell progenitor, GC: germinal center, HSC: hematopoietic stem cell, ILC: innate lymphoid cell, ILCP: innate lymphoid cell progenitor, LMPP: lymphomyelo-primed progenitor, memo: memory, NKP: NK-cell progenitor.

To identify the normal expression pattern for all 20 TALE homeobox genes in early hematopoiesis and lymphopoiesis we analyzed several public datasets. Dataset GSE69239 contains RNA-seq data for HSC, LMPP, CLP, BCP and T-cell progenitors of the double negative (DN) and double positive (DP) stages in addition to mature single positive (SP) CD4- and CD8-positive T-cells [28]. Stages of B-cell development were analyzed using gene expression profiling dataset GSE56315, and mature lymphocytes from peripheral blood via dataset GSE72642 [26,27]. ILCPs in addition to mature ILCs were analyzed using RNA-seq datasets GSE90834 and GSE112591, respectively [29,30]. The applied cutoffs to discriminate positive and negative expression levels were adopted from our previous studies [6,18,20]. The screening results are shown in S1 Fig and summarized in Fig 1. Together, we detected the expression of eleven TALE homeobox genes in the analyzed hematopoietic entities and termed their assembled signature TALE-code.

We found TALE homeobox gene activities in all analyzed lineages and stages. The numbers of expressed genes in single entities ranged from one to eight. TGIF genes were expressed in all stages analyzed, and PKNOX1 expression was detected in all lineages except that of the ILCs. Thus, ILCs only expressed TGIF genes while the activities of MEIS3 and MEIS4 were restricted to the B-cell lineage. Finally, MEIS1 and PBX1 were only expressed in progenitors comprising HSC, LMPP, DN T-cells and ILCPs. Thus, TALE homeobox genes show a specific expression pattern which may underlie regulation of normal cell differentiation in hematopoiesis. Therefore, aberrations of this TALE-code may promote the generation of leukemia and lymphoma.

### Aberrant TALE homeobox gene expression in HL

Here, we additionally analyzed the expression of all TALE homeobox genes in HL patients to compare the results with the established TALE-code. Two public datasets containing gene expression profiling data of 12 (GSE12453) and 29 (GSE39134) classical HL patients were examined [31,32]. In addition, dataset GSE12453 contains expression data from normal developing B-cells as controls. This approach revealed seven overexpressed TALE homeobox genes in HL patients, comprising IRX3, IRX4, MEIS1, MEIS3, PBX1, PBX4, and TGIF1 (S2 Fig). Moreover, detailed analysis of 12 HL patients (GSE12453) showed that all but one (patient cHL7) overexpressed at least one TALE homeobox gene. This observation underlines the oncogenic role of this type of homeobox genes in the pathogenesis of HL. Analysis of these identified seven TALE homeobox genes in HL cell lines using our RNA-seq dataset E-MTAB-7721 and expression profiling dataset GSE115191 revealed significant activity for five genes: IRX3, MEIS1, MEIS3, PBX1, and PBX4 (S3 Fig). Thus, these analyses indicated that particular TALE homeobox genes may play an oncogenic role in the pathogenesis of HL. Affected HL cell lines may serve as models for continued investigation.

For detailed studies we here focused on PBX1. Our data showed that normal expression of PBX1 was restricted to stem and progenitor cells while developing and mature B-cells lacked PBX1 activity (Fig 1). These results were consistent with previous data showing that PBX1 regulates self-renewal of HSCs and determines the potential of myeloid and lymphoid progenitors [38,39]. During B-cell development PBX1 expression is downregulated [40]. Therefore, ectopic expression of PBX1 in differentiating B-cells may promote lymphomagenesis. Accordingly, the fusion gene TCF3-PBX1 reportedly blocks B-cell differentiation and has been detected in pre-B acute lymphoid leukemia (ALL) patients and cognate cell line 697 [41–43].

**Fig 2:**
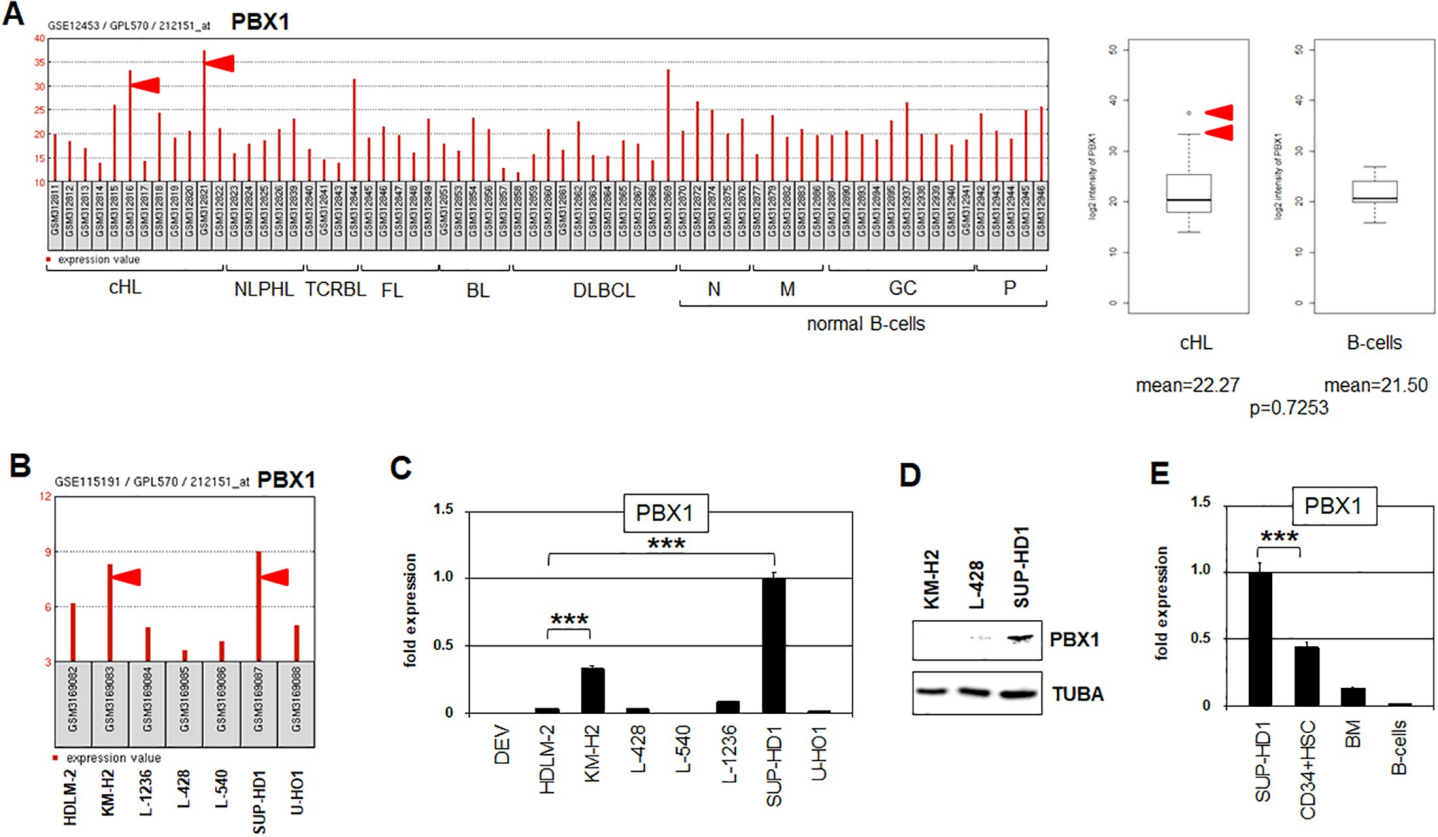
PBX1 expression in HL patients and cell lines. (A) PBX1 expression levels in HL patients according to dataset GSE12453 (left). Statistical analysis of PBX1 expression in cHL patients and normal B-cells using dataset GSE12453. The red arrow heads indicate two patients with highest PBX1 levels. Overall the difference between patients and controls is not significant (right). BL: Burkitt lymphoma, cHL: classical HL, DLBCL: diffuse large B-cell lymphoma, GC: germinal center B-cells, FL: follicular lymphoma, M: memory B-cells, NLPHL: nodular lymphocyte-predominant HL, N: naïve B-cells, P: plasma B-cells, TCRBL: T-cell rich B-cell lymphoma. (B) PBX1 expression levels in HL cell lines according to dataset GSE115191. KM-H2 and SUP-HD1 showed the highest PBX1 levels. (C) RQ-PCR analysis of PBX1 in HL cell lines, confirming enhanced transcript levels in KM-H2 and SUP-HD1. (D) Western blot analysis of PBX1 in HL cell lines showing PBX1 protein in SUP-HD1 but not in KM-H2. TUBA served as loading control. (E) RQ-PCR analysis of PBX1 in HL cell line SUP-HD1 in comparison to primary cells: hematopoietic stem cells (HSC), bone marrow (BM), and peripheral B-cells.

Aberrantly expressed PBX1 was found in about 17% of cHL patients (Fig 2A). Expression analysis of HL cell lines using profiling (GSE115191) and RNA-seq data (E-MTAB-7721) demonstrated elevated PBX1 levels in KM-H2 and SUP-HD1 (Figs 2B and S3). RQ-PCR analysis confirmed enhanced PBX1 expression levels in both cell lines (Fig 2C). Advanced sequence analysis of the RNA-seq data indicated that KM-H2 and SUP-HD1 expressed the splicing variant PBX1a (S4 Fig). Western blot analysis showed PBX1 protein in SUP-HD1 but not in KM-H2 (Fig 2D), indicating post-transcriptional repression in the latter. Finally, RQ-PCR analysis of PBX1 in SUP-HD1 in comparison to primary hematopoietic cells demonstrated even lower levels in HSCs and the bone marrow and confirmed its absence from peripheral B-cells (Fig 2E). Therefore, we subsequently used the cell line SUP-HD1 as model for aberrant PBX1 overexpression to study upstream and downstream factors.

### PBX1 is activated by genomic aberration in HL cell lines

Chromosomal and genomic aberrations widely contribute to gene deregulation in cancer including HL. To analyze if the PBX1 locus at chromosomal position 1q23 is rearranged or shows copy number alterations in KM-H2 and/or SUP-HD1 we performed cytogenetic analysis and genomic profiling. The karyotypes of the analyzed cell lines were as follows:

KM-H2: <3n>der(X)der(Y), -X, -1, -1, -2, -2, -3, -4, -5, -5, -6, -8, -9, -9, -10, -10, -10, -11, -12, -13, -14, -15, -15, -16, -16, -18, -19, -20, -21, -22, +25mar, add(1)(q32), add(3)(q26), add(3)(q26), del(4)(q26q31), i(4q), add(5)(p11), add(6)(q14), add(7)(p14), add(7)(q34), del(7)(q31), add(8)(q24), del(8)(p12), del(11)(q23), i(13q), der(13)t(11;13)(p13;q13), add(14)(p12)x2, del(17)(p12), i(18q).

SUP-HD1: <2n>X, -Y, +5, +8, +9, -13, +19, +mar, der(1)t(1;1)(p34;q12.2), del(2)(p23), ins(2;8)t(p2?3;??), add(4)(p15), add(4)(q23), add(5)(p1?1), i(5p), der(5)t(5;15)(p10;q10), del(6)(p23), der(6)i(6)(q10)der(6)t(6;9)(q2?5;p2?1), der(8)add(8)(p11)add(8)(q2?4), der(8)t(8;12)(p22;?p12), del(9)(q3?2), der(9)t(9;12)(q31;?p12), i(9q), der(11)t(4;11)(?p15;p15), del(11)(q22.2), add(12)(p13), i(14q), der(15)t(2;15)(?p23;q26), i(18q), der(19)del(19)(p12)add(19(q13), del(21)(q22), der(22)t(13;22)(q31;q22.3).

**Fig 3:**
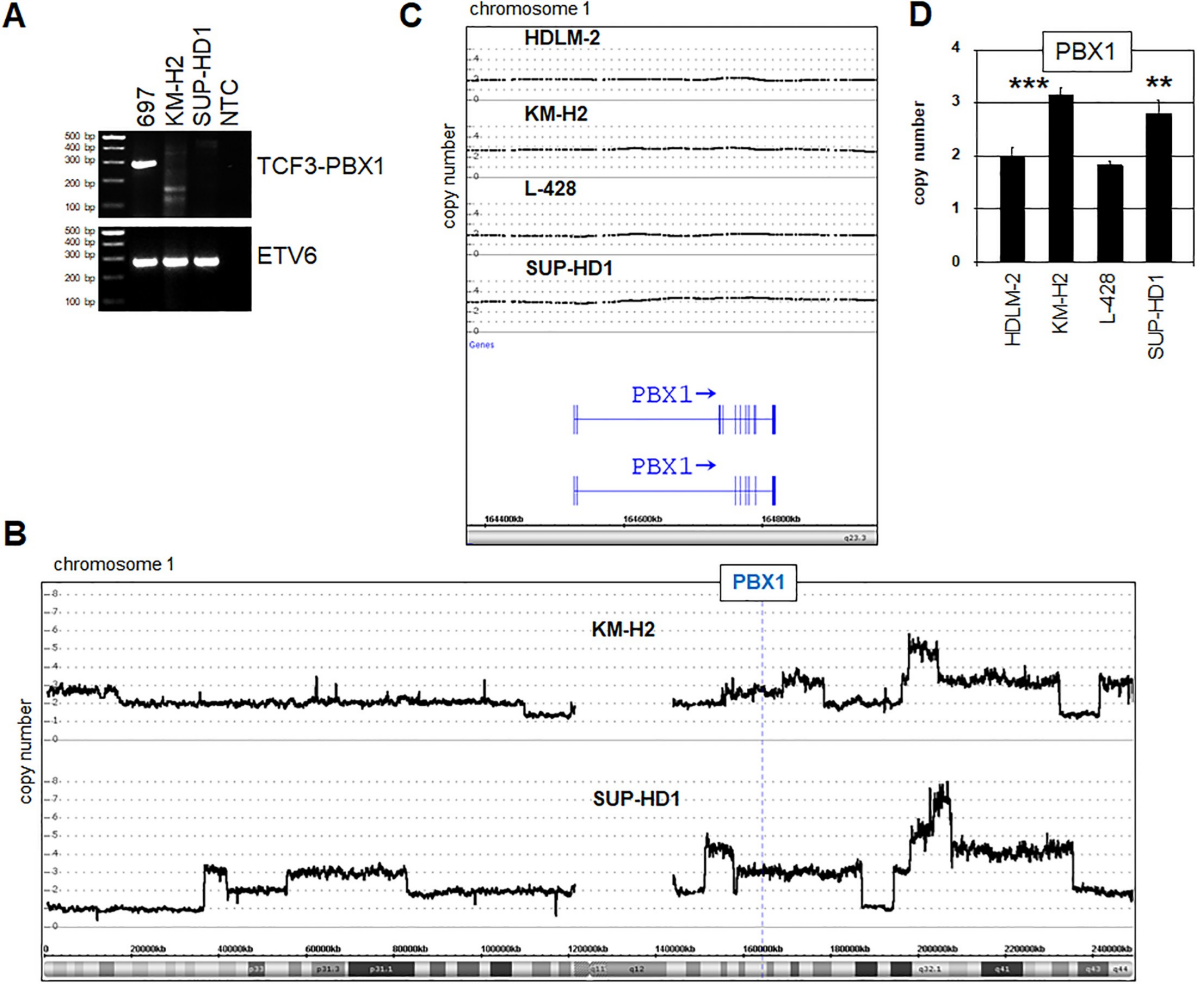
Genomic analyses of the PBX1 locus. (A) RT-PCR analysis of fusion gene TCF3-PBX1 in HL cell lines KM-H2 and SUP-HD1 in addition to pre-B ALL cell line 697 serving as positive control. ETV6 served as control for used cDNA. (B,C) Genomic profiling data of HL cell lines were used to perform copy number analysis for PBX1 located at 1q23. KM-H2 and SUP-HD1 showed three copies while HDLM-2 and L-428 contained two copies. (D) Copy number determination of PBX1 in HDLM-2, KM-H2, L-428 and SUP-HD1 by RQ-PCR.

Karyotypes of both cell lines bore several rearrangements, albeit none at chromosomal band 1q23. RT-PCR analysis detected the pre-B ALL associated fusion gene TCF3-PBX1 in B-cell line 697 which was absent from both HL cell lines KM-H2 and SUP-HD1, excluding the presence of chromosomal aberration t(1;19)(q23;p13), consistent with the cytogenetic findings (Fig 3A). However, analysis of our profiling data revealed a genomic gain at position 1q23 exclusively in these two HL cell lines (Fig 3B and 3C). These genomic gains at PBX1 were confirmed by RQ-PCR analysis (Fig 3D), offering an explanation for enhanced PBX1 transcript levels in HL cell lines KM-H2 and SUP-HD1.

### PBX1 activates oncogene NFIB in HL

**Fig 4:**
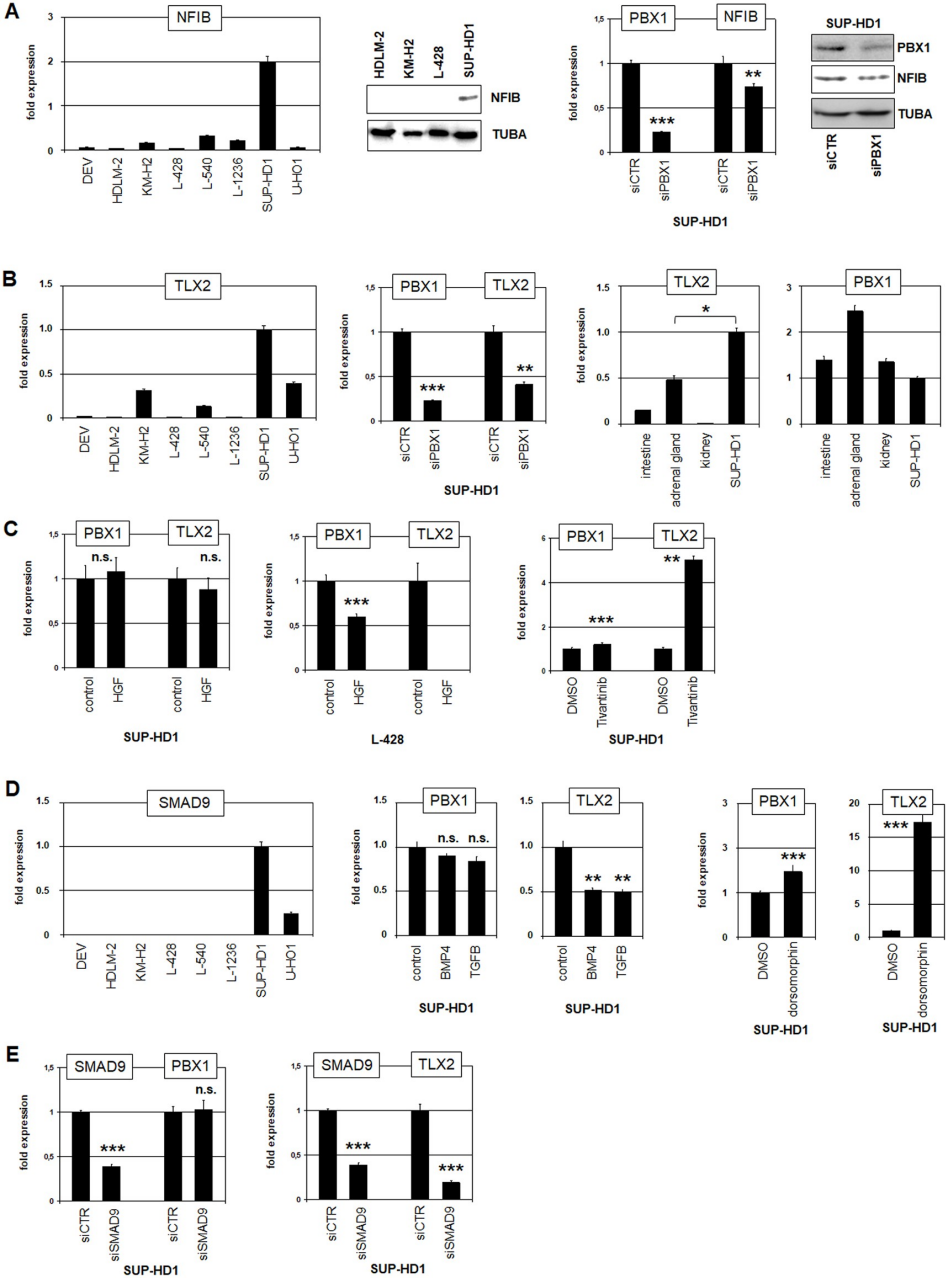
Analysis of PBX1 target genes NFIB and TLX2. (A) RQ-PCR analysis of NFIB in HL cell lines, confirming enhanced transcript levels in SUP-HD1 (left). Western blot analysis of NFIB in HL cell lines showed prominent NFIB expression in SUP-HD1 (middle). SiRNA-mediated knockdown of PBX1 in SUP-HD1 cells and subsequent RQ-PCR and Western blot analysis, showing reduced PBX1 and NFIB expression (right). (B) RQ-PCR analysis of TLX2 in HL cell lines, demonstrating enhanced transcript levels in SUP-HD1 (left). SiRNA-mediated knockdown of PBX1 in SUP-HD1 cells and subsequent RQ-PCR analysis showed reduced PBX1 and TLX2 expression (middle). RQ-PCR analyses of TLX2 and PBX1 in HL cell line SUP-HD1 in comparison to primary cells from intestine, adrenal gland and kidney (right). (C) RQ-PCR analyses of PBX1 and TLX2 in HL cell lines SUP-HD1 and L-428 treated with HGF or MET-inhibitor tivantinib, indicating that HGF-signalling inhibits expression of PBX1 and TLX2. (D) RQ-PCR analysis of SMAD9 in HL cell lines, demonstrating enhanced transcript levels in SUP-HD1 (left). RQ-PCR analysis of PBX1 and TLX2 of SUP-HD1 cells treated with BMP4 or TGFb (middle), or with BMP-receptor-inhibitor dorsomorphin (right) demonstrated that BMP-signalling inhibited PBX1 and TLX2 expression. (E) RQ-PCR analyses of SMAD9 and PBX1 in HL cell line SUP-HD1 treated for siRNA-mediated knockdown of SMAD9 showed reduced expression of SMAD9 and TLX2 but not of PBX1.

We performed comparative expression profiling analyses to identify downstream genes regulated by PBX1 in HL. Accordingly, we examined two datasets (GSE12453 and GSE39134) which contain gene data of classical HL patients, using the online tool GEOR to calculate the top 250 genes showing the most significant differences in their expression levels between PBX1-high and PBX1-low patients. This procedure revealed significant coexpression of PBX1 with NFIB in both datasets (S5 Fig). Analysis of NFIB expression by RNA-seq data of 100 leukemia-lymphoma cell lines (E-MTAB-7721) and RQ-PCR of eight HL cell lines revealed enhanced NFIB transcription in SUP-HD1 cells (Figs 4A and S6). Advanced RNA-seq data analysis showed that SUP-HD1 expressed the large splicing form NFIB-L (S4 Fig). Western blot analysis confirmed NFIB expression in SUP-HD1 at the protein level while controls tested negative (Fig 4A). SiRNA-mediated knockdown of PBX1 in SUP-HD1 resulted in reduced expression levels of NFIB, demonstrating that PBX1 activates NFIB transcription in this HL cell line (Fig 4A).

NFIB is a member of the NFI gene family which contains four members encoding TFs with developmental and oncogenic potential [44]. Interestingly, RNA-seq data analysis demonstrated that remaining family members NFIA, NFIC and NFIX also showed raised expression levels in HL cell lines (S6 Fig), a finding confirmed by expression profiling of HL patients (S7 Fig). We concluded that these NFI family genes represent novel candidate oncogenes implicated in HL pathogenesis.

### PBX1 and SMAD9 activate NKL homeobox gene TLX2 in HL

To identify additional gene candidates located downstream or upstream of PBX1 in HL we analyzed expression profiling data of HL cell lines obtained from dataset GSE115191 (S1 Table). We compared expression data from SUP-HD1 with those of five controls (HDLM-2, L-428, L-540, L-1236, U-HO1). Inspection of the at least 5-fold differentially expressed genes revealed PBX1 and NFIB in addition to TLX2, HGF and SMAD9 which were selected for more detailed examinations.

TLX2 encodes an NKL homeobox gene previously found to be implicated in both T-ALL and HL [6,18]. Interestingly, its close relative TLX1 is a direct target gene of PBX1 in developing spleen [45]. However, TLX1 remained silent in PBX1-expressing SUP-HD1 cells excluding this embryonal relationship from HL (S6 Fig). Therefore, we speculated whether TLX2 may represent an alternative target gene of PBX1. Analysis of TLX2 expression in HL cell lines by RNA-seq (E-MTAB-7721) and RQ-PCR demonstrated enhanced expression in SUP-HD1 correlating with PBX1 (Figs 4B and S6). Furthermore, siRNA-mediated knockdown of PBX1 in SUP-HD1 cells resulted in reduced TLX2 expression levels, supporting that TLX2 represents an additional target gene activated by PBX1 in HL (Fig 4B). Finally, we performed RQ-PCR analysis of TLX2 and PBX1 in primary adrenal gland and intestine cells which reportedly express TLX2 [46]. Our results revealed correlated expression levels of TLX2 and PBX1 in these tissues, and that SUP-DH1 cells expressed higher levels of TLX2 (Fig 4B). Thus, TLX2 is a target gene of PBX1 in HL and is overexpressed in SUP-HD1 when compared to physiological levels.

HGF (hepatocyte growth factor) and SMAD9 are components of HGF-MET- and BMP-signalling pathways, respectively. We speculated whether these pathways might contribute to aberrant expression of PBX1 and/or TLX2 in HL. The HGF-MET-pathway has been implicated in the pathogenesis of HL, supporting a potential role for HGF-signalling in this context [47]. Both, HGF ligand and its receptor the tyrosine kinase MET were highly expressed in SUP-HD1 cells as demonstrated by RNA-seq data analysis (S6 Fig). However, treatment of SUP-HD1 and HL cell line L-428 which express only MET highly with HGF or MET-inhibitor tivantinib showed that HGF-signalling repressed both, PBX1 and its target TLX2 (Fig 4C). Despite its inhibitory activity, the HGF-MET-pathway did not silence PBX1 expression in SUP-HD1 cells.

SMAD9 operates as a repressor of the BMP-signalling pathway [48]. SMAD9 expression level was high in SUP-HD1 only, according to both, RNA-seq data and RQ-PCR analyses (Figs 4D and S6). However, treatment of SUP-HD1 cells with BMP4 or TGFb resulted in slightly reduced PBX1 and strongly reduced TLX2 expression (Fig 4D), indicating a repressive impact by these related pathways. Moreover, treatment with BMP-receptor inhibitor dorsomorphin enhanced TLX2 expression strongly and PBX1 only slightly (Fig 4D). Thus, BMP-signalling inhibited TLX2 expression in HL without implication of PBX1. Accordingly, siRNA-mediated knockdown of SMAD9 in SUP-HD1 resulted in reduced TLX2 expression while PBX1 remained unchanged (Fig 4E). Thus, enhanced expression of SMAD9 in SUP-HD1 inhibits BMP-signalling and thereby supports TLX2 transcription.

### Functional analysis of NKL homeobox gene TLX2 in HL

**Fig 5:**
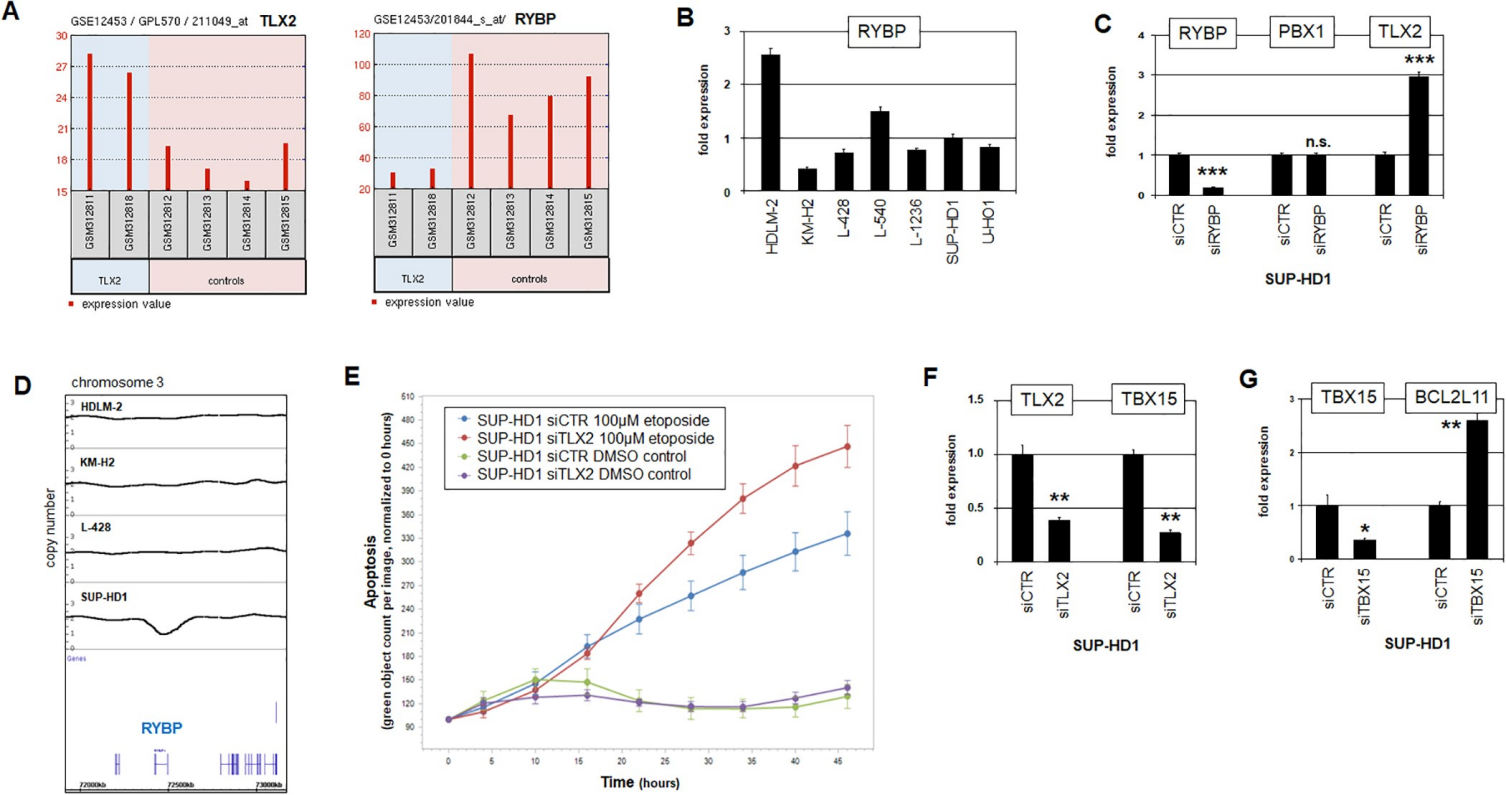
Analysis of TLX2, RYBP and TBX15. (A) Comparative expression profiling analysis of HL patients using dataset GSE12453. Patients with elevated expression levels of TLX2 showed significantly reduced expression levels of inhibitor RYBP (p = 0.000106). (B) RQ-PCR analysis of RYBP in HL cell lines. (C) RQ-PCR analyses of RYBP, PBX1 and TLX2 in HL cell line SUP-HD1 treated for siRNA-mediated knockdown of RYBP showed reduced expression of RYBP and elevated expression of TLX2 while PBX1 expression level were unchanged. (D) Genomic profiling data of HL cell lines were used to perform copy number analysis for RYBP located at 3p13. SUP-HD1 showed a deletion while HDLM-2, KM-H2 and L-428 showed the wild type configuration. (E) Live-cell imaging analysis of SUP-HD1 cells treated for siRNA-mediated knockdown of TLX2 in addition to 100 μM etoposide, demonstrating enhanced apoptosis in the presence of etoposide after TLX2 reduction. (F) RQ-PCR analyses of TLX2 and TBX15 in HL cell line SUP-HD1 treated for siRNA-mediated knockdown of TLX2. Reduced expression levels of both genes indicated that TLX2 activated TBX15. (G) RQ-PCR analyses of TBX15 and BCL2L11 in HL cell line SUP-HD1 treated for siRNA-mediated knockdown of TBX15. Reduced expression levels of TBX15 and elevated levels of BCL2L11 indicated that TBX15 inhibited BCL2L11.

Analysis of PBX1 target gene TLX2 was performed using the online tool GEOR for expression profiling data from HL patients (GSE12453). Comparison of TLX2-high with TLX2-low patients demonstrated significantly reduced RYBP expression in patients with enhanced TLX2 activity (Fig 5A). RYBP encodes an inhibitory component of the polycomb repressor complex (PRC)1 which regulates several homeobox genes [49,50]. Analyses of RYBP expression by RNA-seq data (E-MTAB-7721) and RQ-PCR demonstrated differential transcript levels in HL cell lines (Figs 5B and S6). Reduction of RYBP by siRNA-mediated knockdown in SUP-HD1 resulted in elevated TLX2 expression levels while that of PBX1 remained unchanged (Fig 5C). These results indicated that this repressor mediates inhibition of NKL homeobox gene TLX2 but not of TALE homeobox gene PBX1. Interestingly, genomic profiling data of SUP-HD1 showed copy number alterations specifically at the RYBP locus at chromosomal position 3p13 which may underlie aberrant RYBP transcription in this cell line (Fig 5D).

To analyze the pathological function of TLX2 we performed live-cell imaging of SUP-HD1 cells treated for siRNA-mediated knockdown of this gene. While this treatment alone showed no effect as compared to the control, simultaneous treatment with apoptosis-inducer etoposide activated apoptosis in cells with reduced TLX2 levels significantly more strongly (Fig 5E). Thus, TLX2 supports cell survival in HL cells. To identify corresponding target genes regulated by TLX2 in HL we performed expression profiling analysis of SUP-HD1 cells treated for siRNA-mediated knockdown of TLX2 (S2 Table). Subsequent inspection of the at least 2-fold differentially expressed genes highlighted TLX2 in addition to TBX15. TBX15 is a T-box TF and implicated in regulation of cell differentiation and apoptosis [51,52]. RQ-PCR analysis of treated SUP-HD1 cells confirmed the TLX2 knockdown and demonstrated concomitantly reduced TBX15 transcription, supporting that TLX2 activated TBX15 expression (Fig 5F). Furthermore, siRNA-mediated knockdown of TBX15 in SUP-HD1 cells boosted expression of pro-apoptotic BCL2L11/BIM (Fig 5G). Collectively, these data show that TLX2 inhibits apoptosis by activation of TBX15 and subsequent suppression of BCL2L11.

### Cooperation between PBX1 and HOXB9

**Fig 6:**
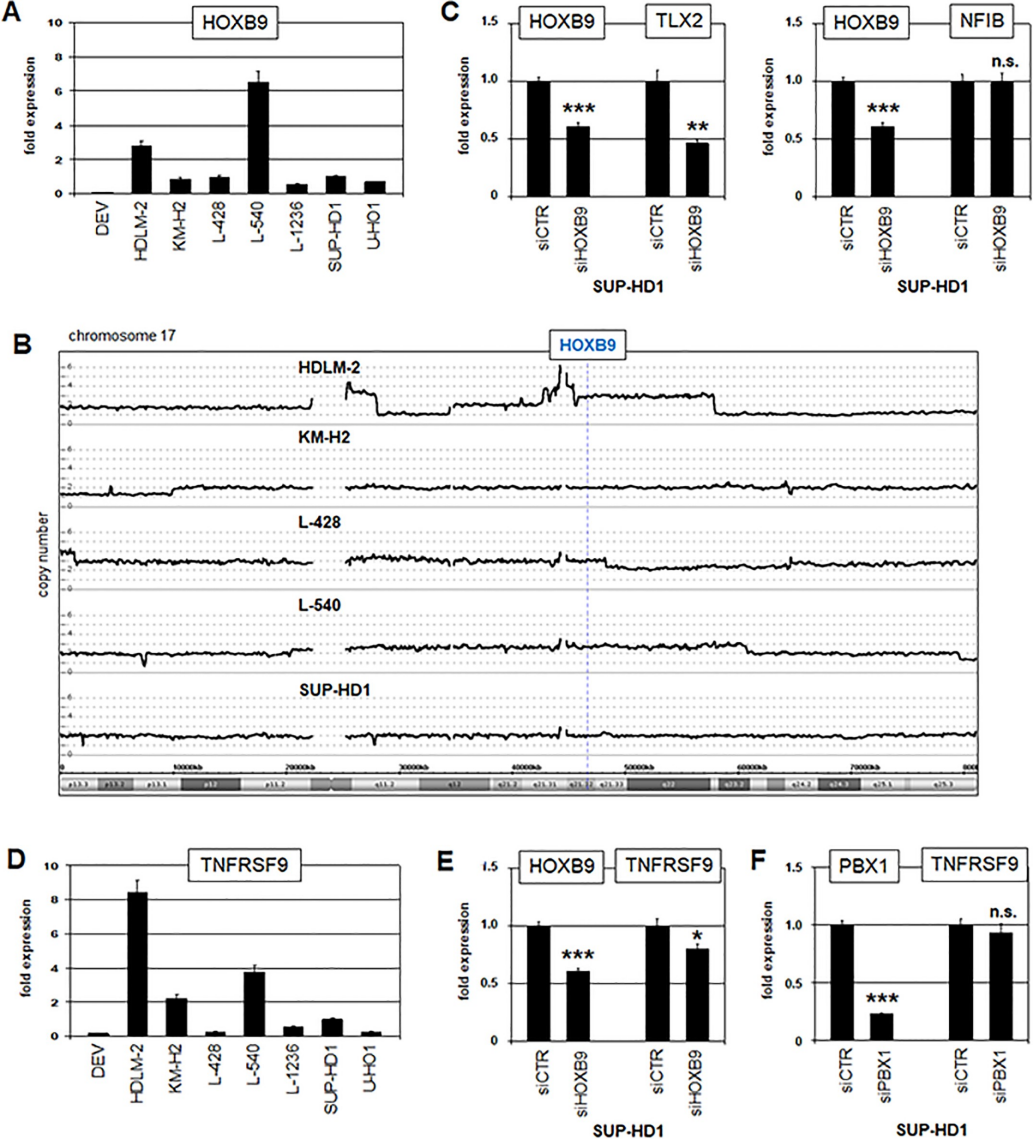
Cooperation of PBX1 and HOXB9. (A) RQ-PCR analysis of HOXB9 in HL cell lines, demonstrating enhanced transcript levels in HDLM-2 and L-540. (B) Genomic profiling data of HL cell lines HDLM-2, KM-H2, L-428, L-540 and SUP-HD1, showing copy number states for HOXB9 at chromosomal position 17q21. (C) RQ-PCR analyses of HOXB9 and TLX2 (left) and of HOXB9 and NFIB (right) in HL cell line SUP-HD1 treated for siRNA-mediated knockdown of HOXB9. The results indicated that HOXB9 activated TLX2 but not NFIB. (D) RQ-PCR analysis of TNFRSF9 in HL cell lines, demonstrating enhanced transcript levels in HDLM-2 and L-540. Please note the correlation of TNFRSF9 and HOXB9 expressio levels. (E) RQ-PCR analyses of HOXB9 and TNFRSF9 in HL cell line SUP-HD1 treated for siRNA-mediated knockdown of HOXB9, indicating that HOXB9 activated TNFRSF9 expression. (F) RQ-PCR analyses of PBX1 and TNFRSF9 in HL cell line SUP-HD1 treated for siRNA-mediated knockdown of PBX1, indicating that PBX1 did not regulate TNFRSF9 expression.

PBX1, in addition to other TALE homeodomain proteins, cooperates with certain TALE and HOX proteins to regulate their target genes [53,54]. In a previous study, we identified aberrant overexpression of homeobox gene HOXB9 in HL cell lines, endorsing this gene as a candidate cooperation patner of PBX1 in this malignancy [55]. Here, analysis of HOXB9 expression using datasets GSE12453 and GSE39134 confirmed elevated levels in HL patients (S7 Fig). When HOXB9 expression was quantified in HL cell lines by RNA-seq and RQ-PCR analyses, the highest levels were detected in HDLM-2 and L-540 (Figs 6A and S6). Copy number analysis for HOXB9 in HL cell lines by genomic profiling showed a gain of its locus at 17q21 for HDLM-2 and L-540 but also for L-428 (Fig 6B). These results indicated copy number gains and additional unknown factors enhancing HOXB9 expression in HL.

To analyze a potential cooperation between PBX1 and HOXB9 in HL we performed siRNA-mediated knockdown of HOXB9 in SUP-HD1 and examined identified PBX1 target genes. This experiment showed that HOXB9 supported activation of TLX2 but not of NFIB (Fig 6C). To identify additional HOXB9 target genes we performed expression profiling analysis of HDLM-2 cells treated for siRNA-mediated knockdown of HOXB9 (S3 Table). Inspection of these data revealed TNFRSF9 (TNF receptor superfamily member 9). This gene has been implicated in the immune escape of malignant HL cells, showing to be a clinically relevant target for this disease [56]. RNA-seq and RQ-PCR analyses of TNFRSF9 demonstrated corresponding expression levels with HOXB9 in HL cell lines (Figs 6D and S6). Furthermore, RQ-PCR analysis of SUP-HD1 showed reduced TNFRSF9 expression after HOXB9 knockdown, confirming that HOXB9 activated TNFRSF9 transcription in HL (Fig 6E). However, knockdown of PBX1 left TNFRSF9 expression unperturbed in these cells (Fig 6F), contradicting involvement of PBX1 in TNFRSF9 regulation. Thus, homeobox gene TLX2 was regulated by both PBX1 and HOXB9, and NFIB by PBX1 without HOXB9 while TNFRSF9 was regulated by HOXB9 without PBX1. These results show distinct differences in cooperative gene regulation between TALE homeodomain protein PBX1 and HOXL subclass member HOXB9 in HL cells.

### Analysis of PBX1 network genes in normal lymphopoiesis

Extended analysis of PBX1 expression in a panel of 30 immune cell entities using public dataset GSE107011 showed the highest transcript levels in progenitors (S8A Fig) [57], supporting our screening data and the described role of this gene in manifestation of stemness and regulation of lineage differentiation [38,39]. Interestingly, NFIB showed a similar expression pattern (S8A Fig). Coexpression of PBX1 and NFIB was also detected in RNA-seq dataset GSE69239, showing peak levels in HSCs and undifferentiated DN T-cells (S8B Fig). Together, these data may indicate similar functions for NFIB in immune cell differentiation as described for PBX1.

Analysis of TLX2 expression in dataset GSE69239 showed activity in DN T-cells as reported previously (S8B Fig) [18]. Coexpression of TLX2 and PBX1 in these cells may reflect the regulatory connection we identified between PBX1 and TLX2 in HL, and underlines the physiological significance of this interplay. Furthermore, downregulation of RYBP in DN T-cells coincided with increased TLX2 expression levels (S8B Fig), indicating a requirement for RYBP silencing for TLX2 activity in the physiological hematopoietic context. Collectively, the aberrant network in HL identified here, consisting of PBX1, NFIB, TLX2 and RYBP may have emerged following aberrant activation of PBX1, downregulation of RYBP, and subsequent deregulation of genes normally active in differentiation of stem and progenitor cells. Therefore, this network has the potential to disturb the process of B-cell differentiation in HL.

## Discussion

**Fig 7:**
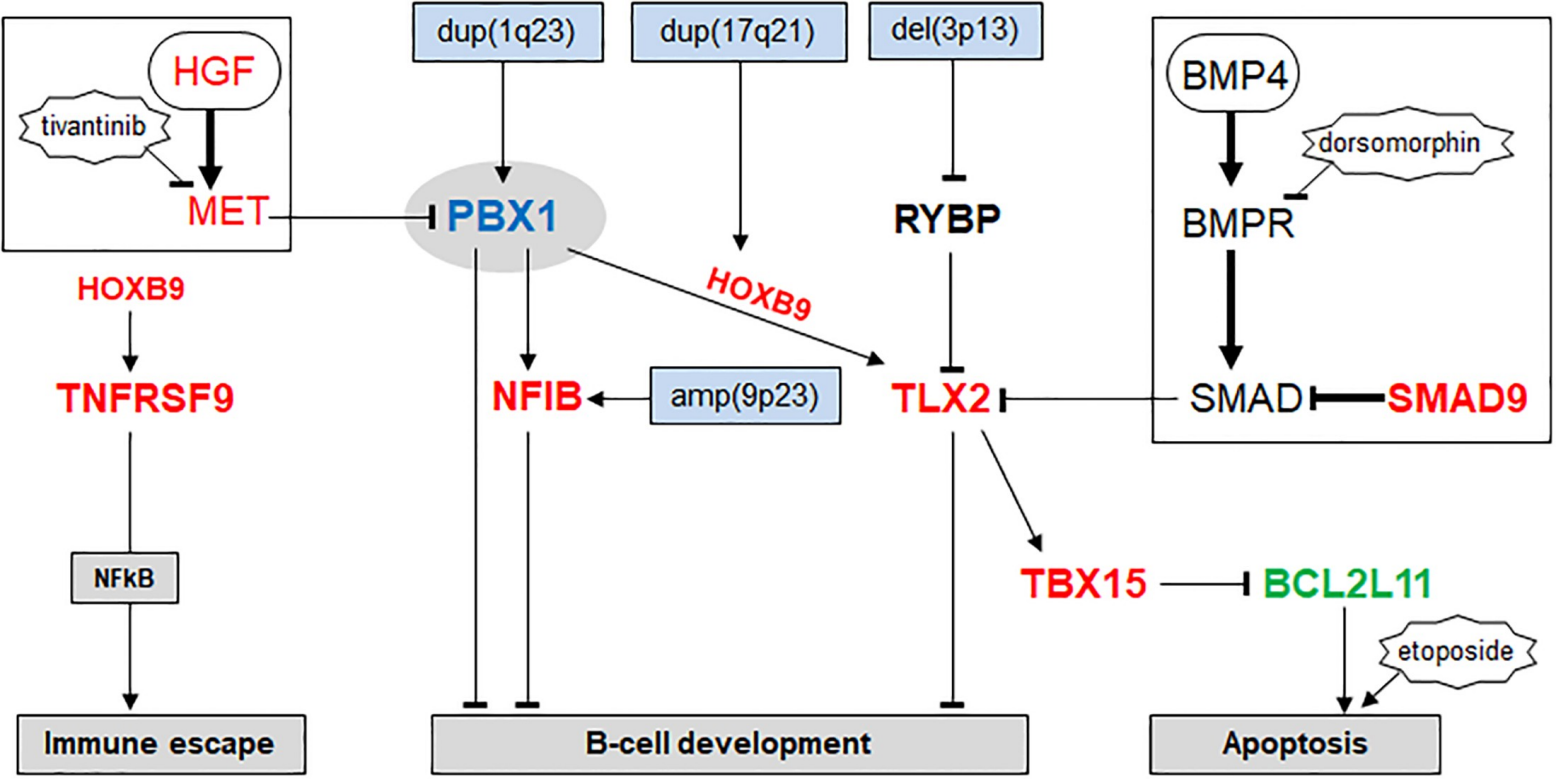
Gene regulatory network around PBX1 in HL. This diagram summarizes the results of the study. Chromosomal rearrangements (blue boxes) deregulate PBX1, RYBP and NFIB. PBX1 activates NFIB and TLX2 but not TNFRSF9. HOXB9 activates TNFRSF9 without PBX1, and TLX2 in cooperation with PBX1. TBX15 is a target gene of TLX2 and inhibits pro-apoptotic gene BCL2L11. HGF-signalling inhibits PBX1 and BMP-signalling inhibits TLX2 expression (both pathways are in black boxes). SMAD9 operates as inhibitor of the BMP-pathway.

Comprehensive expression analysis of TALE homeobox genes in early hematopoiesis and lymphopoiesis revealed a gene activity pattern for the included entities which we termed the TALE-code (Fig 1). This code epitomizes physiological gene expressions for 11 of 20 described TALE homeobox genes and serves as tool for identification and evaluation of deregulated class members in lymphoid malignancies. These data demonstrate PBX1 activity in hematopoietic stem and progenitor cells and correspond to reports showing that PBX1 regulates self renewal and lineage choice in HSCs and myeloid and lymphoid compartments [38,39]. Of note, we were unable to analyze TALE homeobox gene activity in progenitor entities CILP and NKP due to the lack of corresponding expression data. However, the report that PBX1 activates NK-cell master gene NFIL3 may indicate that PBX1 is active in NKPs as well [58]. About 17% of HL patients we analyzed and two out of eight HL cell lines transcribed PBX1 aberrantly. In addition, our investigations revealed an aberrant gene network around PBX1 operating in HL (Fig 7). Further expression analyses indicated aberrant reactivation of these genes in undifferentiated hematopoietic entities including HSC and various progenitors.

PBX1 operates as oncogene in various tumors, including myoepithelioma and ovarian cancer [59,60], and our data now extends its pathogenic role to HL. The oncogenic function of PBX1 may be related to its known activity as a so-called pioneer factor [61]. These factors are mainly active in stem cells and have the potential to activate genes packaged in repressive chromatin. A physiological role of PBX1 in the development of the skeleton, neural crest derived structures, and multiple organs has been shown in knockout mice [62]. Furthermore, the initial development of the spleen depends on PBX1 [45]. In the hematopoietic system, PBX1 is mainly active in stem and progenitor cells [38]. Accordingly, the oncogenic activity of fusion gene TCF3-PBX1 in pre-B ALL in blocking B-cell differentiation may be related to physiological downregulation of PBX1 in B-cell development [40,43].

The PBX1 gene is located at chromosomal band 1q23. We identified a correlation between a genomic gain at 1q23 and the aberrant activity of the targeted PBX1 gene in two HL cell lines. Chromosomal aberrations at 1q or 1q23 have also been described in HL patients, suggesting that PBX1 may represent a target gene of these rearrangements in this malignancy [63–65]. In addition to the genomic gain at 1q23, HL cell line SUP-HD1 bears an amplicon at 9p23, hosting NFIB, which may also support its enhanced expression. Interestingly, both genomic aberrations have been described in patients with myeloproliferative neoplasms (MPN) [66]. Therefore, our finding that PBX1 activates NFIB in HL may indicate that this relationship also holds in MPN.

NFIB encodes a basic regulator of cell differentiation implemented in lung, brain, and submandibular gland development [67]. In addition, NFIB plays a role in hematopoiesis as shown for developing megakaryocytes [68]. NFIB is a member of the NFI family which further contains the genes NFIA, NFIC and NFIX [44]. Other members of the NFI family are also involved in hematopoiesis, including NFIX in HSCs [69], and NFIA in erythropoiesis [70]. NFI-group proteins undergo homo- and heterodimerization [67], indicating cooperative modes of activity. All NFI family members are implicated in various cancers, operating as both oncogenes or tumor suppressors [44]. We have shown that all four members of the NFI family were overexpressed in HL cell lines and patients, indicating that these genes play an oncogenic role in the pathogenesis of HL. Of note, SUP-HD1 was the only HL cell line expressing NFIB while lacking NFIX activity. This observation may indicate that their cooperation might have adverse effects on coexpressing malignant HL cells—an assumption which deserves additional investigation. Nevertheless, their physiological impact in developmental processes suggests that aberrant activity of NFI genes in HL interferes with B-cell differentiation.

NKL homeobox gene TLX2 represents an additional identified target gene of PBX1 in HL. TLX2 is normally expressed in neural crest derived cells where it is activated by PBX1 as well [46,71]. Furthermore, normal TLX2 expression has been detected in T-cells of the DN-stage of development while TLX2 is aberrantly activated in subsets of T-ALL patients [18]. Indeed, NKL homeobox genes represent the largest group of oncogenes deregulated in T-ALL [18,72]. This group includes two genes deregulated by aberrantly expressed inhibitors of the BMP-signalling pathway. Overexpression of CHRDL1 mediates activation of MSX1 and overexpression of FSTL1 and SOSTDC1 causes activation of NKX3-2 [73,74]. TLX2 is also regulated by the BMP-pathway as shown in murine mesoderm development and embryonal carcinoma cells [75,76]. Our data showed that deregulation of the BMP-pathway via overexpression of inhibitory SMAD9 contributed to TLX2 activation in HL, demonstrating that aberrations in this pathway underlie deregulation of NKL homeobox genes in both T-ALL and HL.

In splenic organogenesis TALE homeobox gene PBX1 and downstream activated NKL homeobox genes TLX1, NKX2-5 and NKX3-2 create a regulatory network which controls initial development [45]. Aberrant expression of these splenic NKL homeobox genes has been described in T-ALL while TALE homeobox gene PBX1 plays no obvious oncogenic role in this malignancy [46,74,77]. Consistently, PBX1 knockout mice show elevated levels of T-cells, suggesting that PBX1 has an adverse effect in developing T-cells [38]. Thus, in T-ALL we just see aberrant expression of splenic NKL homeobox genes without PBX1 activity while in HL we find aberrant expression of PBX1 without activation of splenic NKL homeobox genes.

Our data indicated tumor suppressor activity of RYBP in HL which thereby contributed to elevated TLX2 expression. Accordingly, the observed deletion of RYBP in SUP-HD1 at 3p13 may be related to common 3p-deletions in hematologic malignancies [78]. RYBP interacts with PRC1 to effect gene repression [49]. Several homeobox genes are regulated by PRC1, including HOXL and NKL homeobox genes [6,50]. However, a previous publication indicated increased RYBP levels in HL which ascribes oncogenic activity [79]. Nevertheless, altered RYBP in addition to the reported potential of PBX1 as pioneer factor may result in chromatin decompaction and subsequent activation of target genes like TLX2 [61].

Furthermore, we identified TBX15 as an activated target gene of TLX2. TBX15 is normally involved in limb development and plays a role in tumorigenesis as well [51,52]. In cancer cells, TBX15 impacts apoptosis which represents a hallmark for HL [21,52]. Moreover, TBX15 is regulated by NFkB which is an additional molecular hallmark for this disease and may thereby contribute to its aberrant activity [21,80]. Thus, deregulated TBX15 expression is intimately connected with basic pathogenic processes in HL.

PBX1 interacts and cooperates with other TALE homeodomain and HOX proteins, including HOXB9 [54,81,82]. Aberrantly expressed HOXB9 has been described both in HL and solid cancer, underlining the widespread oncogenic role of this homeobox gene [55,83]. Here, we analyzed the role of HOXB9 in the regulation of PBX1 target genes identified in this study. Thus, HOXB9 coactivated TLX2 but not NFIB. Furthermore, TNFRSF9 was regulated by HOXB9 but not by PBX1, indicating that a potential cooperation between PBX1 and HOXB9 in HL did not operate invariably. However, due to the complexity of the interactions between these proteins and their DNA-targets, these observed differences remain unresolved and deserve additional examination [84]. Nevertheless, the HOXB9 activated gene TNFRSF9 plays a role in immune escape in HL and may, thus, represent a novel therapeutic target in this malignancy [56].

In conclusion, our proposed TALE-code extends and specifies the list of TFs ascribed a role in controlling normal hematopoiesis. This report endorses the concept of gene codes to allow identification and evaluation of oncogenes. Furthermore, our work deepened the understanding of developmental deregulation in HL. Previous studies demonstrated aberrant downregulation of basic developmental B-cell factors, including PAX5 and EBF1 in HL [22,23]. However, attempted therapeutic reactivation of silenced TFs like PAX5 in this disease revealed the complexity of underlying networks which hindered the success [85]. Here, we added the developmental TFs PBX1 and HOXB9 and their gene network which may deregulate B-cell differentiation in HL via their aberrant upregulation. Targeting of PBX1-HOX interactions by peptide-competition has been applied in various types of cancer [86]. Therefore, this approach may likewise work in HL subsets. However, therapeutic inhibition of deregulated TFs involved in basic development and differentiation may be accompanied by adverse side-effects due to hitherto uncharacterized pleiotropic functions. Therefore, the knowledge of their downstream targets may pave the way for the design of alternative therapeutic approaches.

## Supporting information

S1 Fig TALE screening in early hematopoiesis and lymphopoiesis. Analyses of five public datasets to reveal 20 TALE homeobox gene activities in early lymphopoiesis, T-cell and B-cell development, mature lymphocytes, and mature and progenitor ILCs.
(TIF)

S2 Fig TALE screening in HL patients. Analysis of TALE homeobox genes using expression profiling datasets GSE39134 and GSE12453 revealed seven genes overexpressed in subsets of HL patients (red).
(TIF)

S3 Fig TALE expression in HL cell lines. Expression data of selected TALE homeobox genes using RNA-seq dataset E-MTAB-7721 (above) and expression profiling dataset GSE115191 (below). HL cell lines are indicated.
(TIF)

S4 Fig Splicing forms of PBX1 and NFIB. RNA-seq data analysis using dataset E-MTAB-7721 for cell lines SUP-HD1 and KM-H2 demonstrating expressed splicing forms PBX1a and NFIB-L.
(TIF)

S5 Fig Comparative expression profiling analysis of HL patients. Analysis of datasets GSE12453 and GSE39134 selecting PBX1-high and PBX1-low controls showed significant coexpression of PBX1 and NFIB in HL patients.
(TIF)

S6 Fig Expression analysis of selected genes in cell lines. Analysis of gene expression using RNA-seq dataset E-MTAB-7721. HL cell lines are indicated.
(TIF)

S7 Fig Expression of NFI genes and HOXB9 in HL patients. Gene expression analysis of NFI-family genes NFIA, NFIB, NFIC and NFIX in addition to HOXB9 using datasets GSE12453 and GSE39134. Samples of normal B-cells are indicated.
(TIF)

S8 Fig Activity of PBX1, NFIB, TLX2, and RYBP in immune cells. Gene expression analysis of four selected genes using datasets GSE107011 (left) and GSE69239 (right).
(TIF)

S1 Table Expression profiling data of HL cell lines (GSE115191). Gene expression profiling data for seven HL cell lines are indicated. Line H451U gives medium expression levels for cell lines HDLM-2, L-428, L-540, L-1236 and U-HO1. Line S-H451U gives the differential gene expression levels between SUP-HD1 and H451U.
(XLS)

S2 Table Gene expression profiling data of SUP-HD1. SUP-HD1 was treated for siRNA-mediated knockdown of TLX2 in comparison to a siRNA control. Differential gene expression levels are indicated in the right lane.
(XLS)

S3 Table Gene expression profiling data of HDLM-2. HDLM-2 was treated for siRNA-mediated knockdown of HOXB9 in comparison to a siRNA control. Differential gene expression levels are indicated in the right lane.
(XLS)

S1 File (TIF)
