# Supplementary figures and images for: Establishment of the TALE-code reveals aberrantly activated homeobox gene PBX1 in Hodgkin lymphoma
Source: PLoS One. 2021 Feb 4;16(2):e0246603. doi: 10.1371/journal.pone.0246603 (PMC7861379; doi:10.1371/journal.pone.0246603)

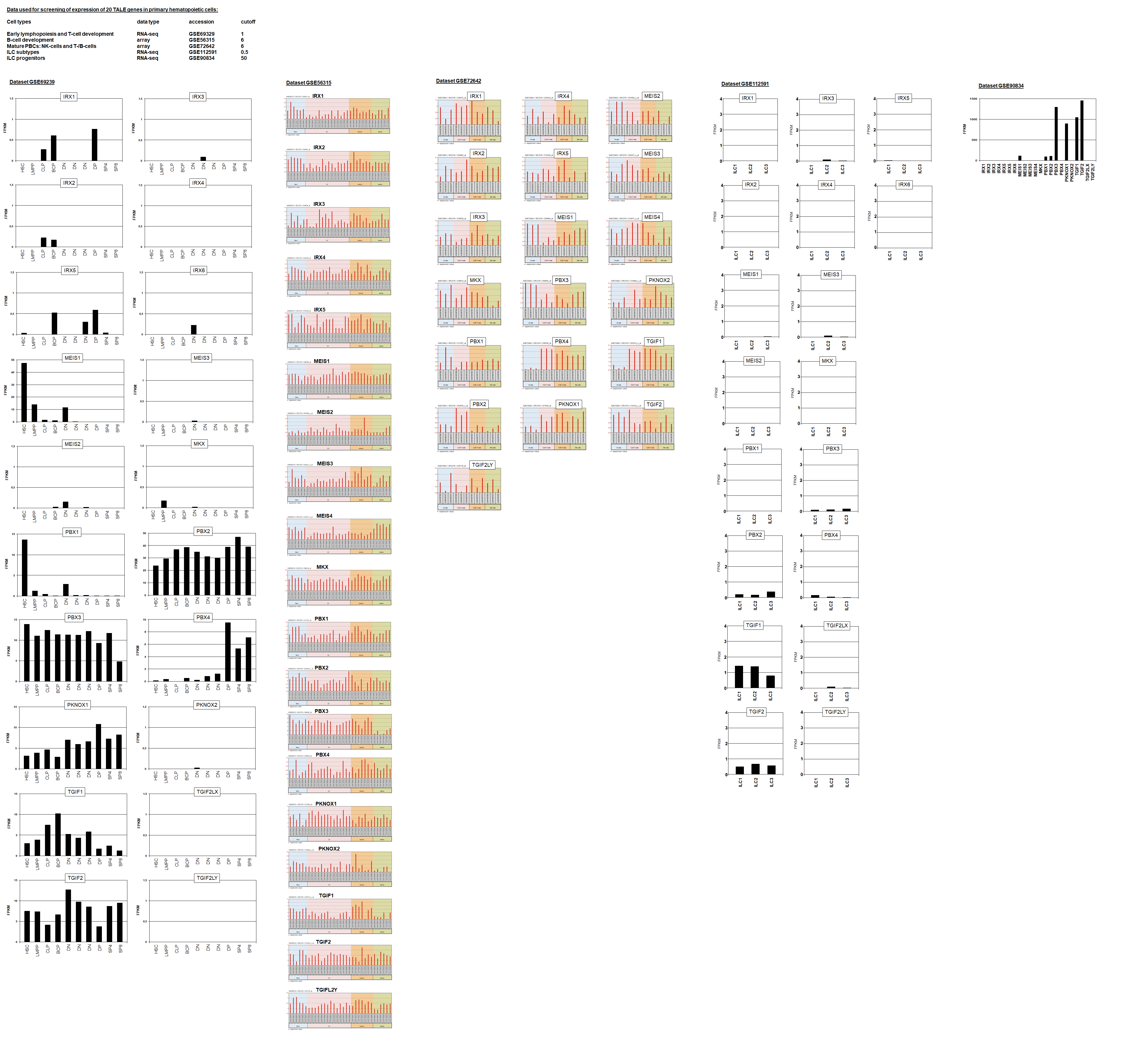

Supplement: S1 Fig — Analyses of five public datasets to reveal 20 TALE homeobox gene activities in early lymphopoiesis, T-cell and B-cell development, mature lymphocytes, and mature and progenitor ILCs. (TIF) [file pone.0246603.s001.tif]

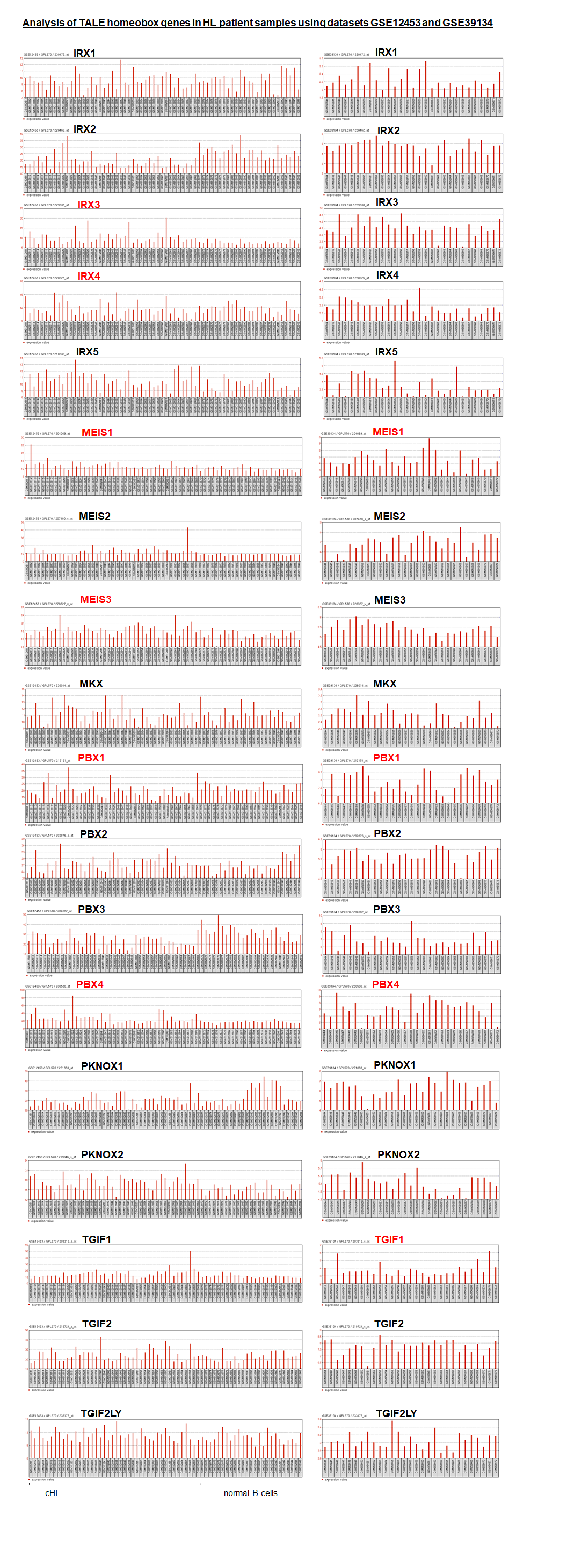

Supplement: S2 Fig — Analysis of TALE homeobox genes using expression profiling datasets GSE39134 and GSE12453 revealed seven genes overexpressed in subsets of HL patients (red). (TIF) [file pone.0246603.s002.tif]

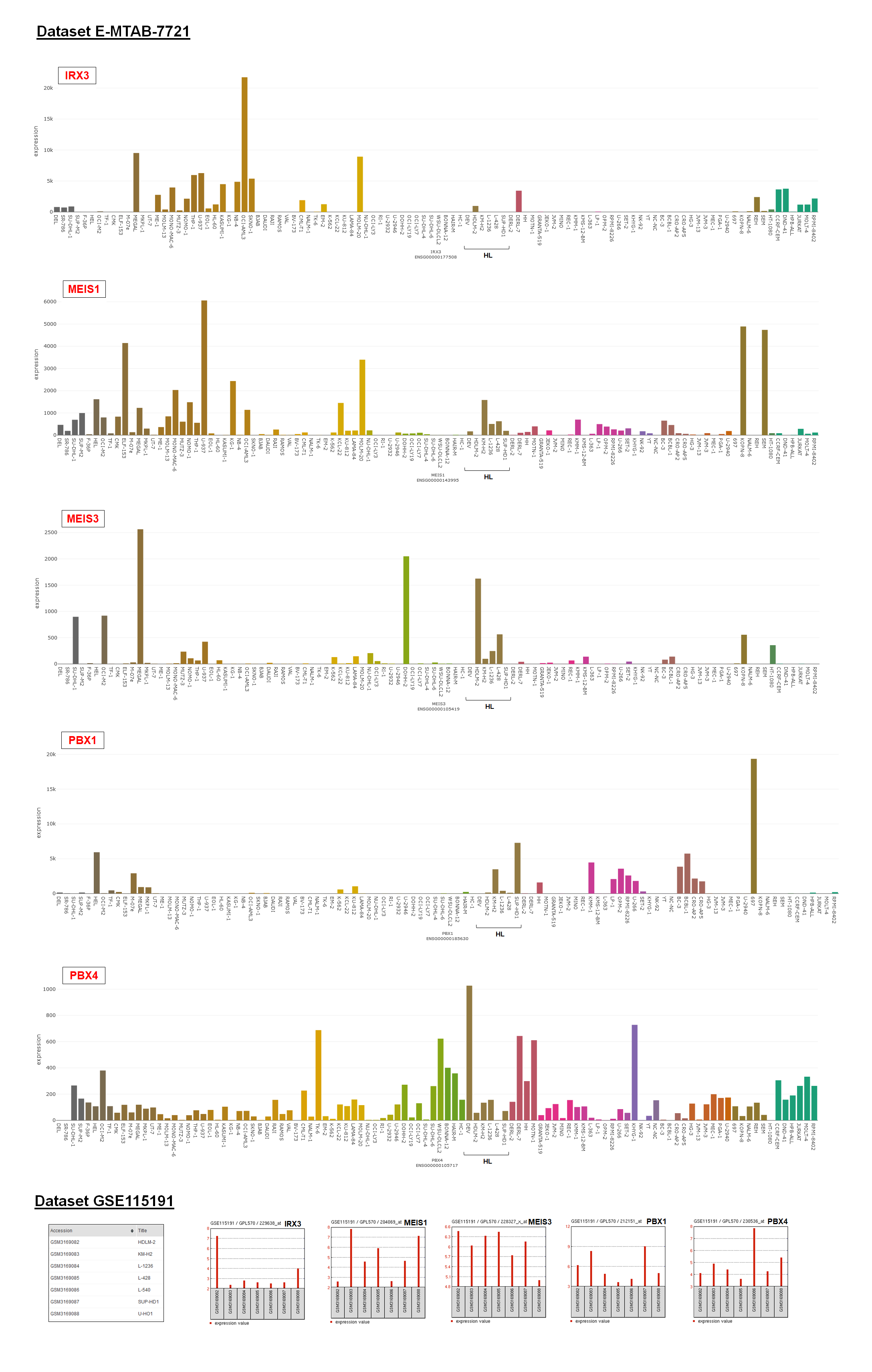

Supplement: S3 Fig — Expression data of selected TALE homeobox genes using RNA-seq dataset E-MTAB-7721 (above) and expression profiling dataset GSE115191 (below). HL cell lines are indicated. (TIF) [file pone.0246603.s003.tif]

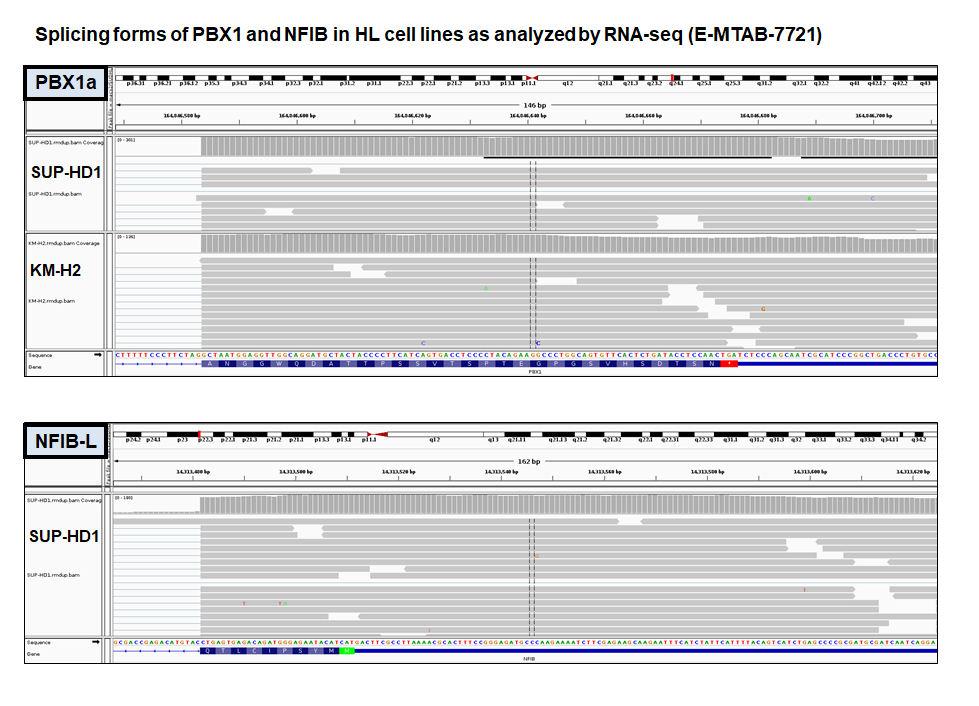

Supplement: S4 Fig — RNA-seq data analysis using dataset E-MTAB-7721 for cell lines SUP-HD1 and KM-H2 demonstrating expressed splicing forms PBX1a and NFIB-L. (TIF) [file pone.0246603.s004.tif]

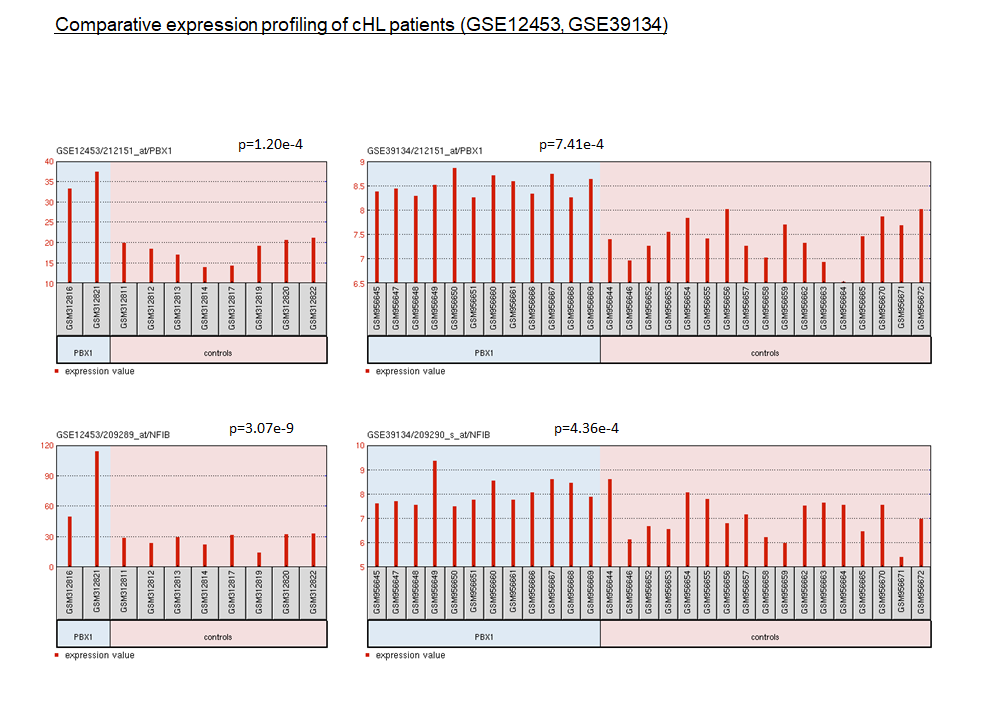

Supplement: S5 Fig — Analysis of datasets GSE12453 and GSE39134 selecting PBX1-high and PBX1-low controls showed significant coexpression of PBX1 and NFIB in HL patients. (TIF) [file pone.0246603.s005.tif]

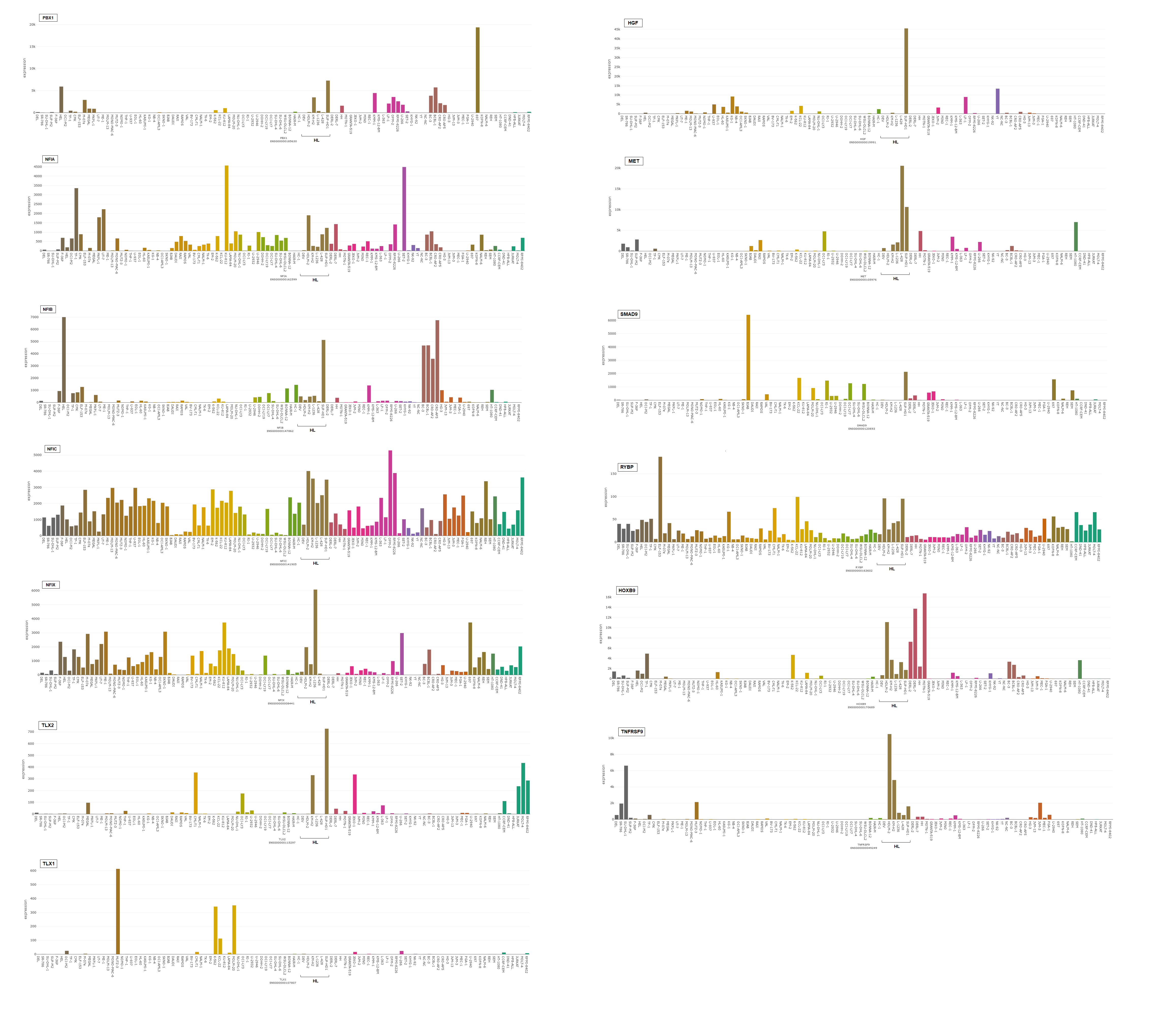

Supplement: S6 Fig — Analysis of gene expression using RNA-seq dataset E-MTAB-7721. HL cell lines are indicated. (TIF) [file pone.0246603.s006.tif]

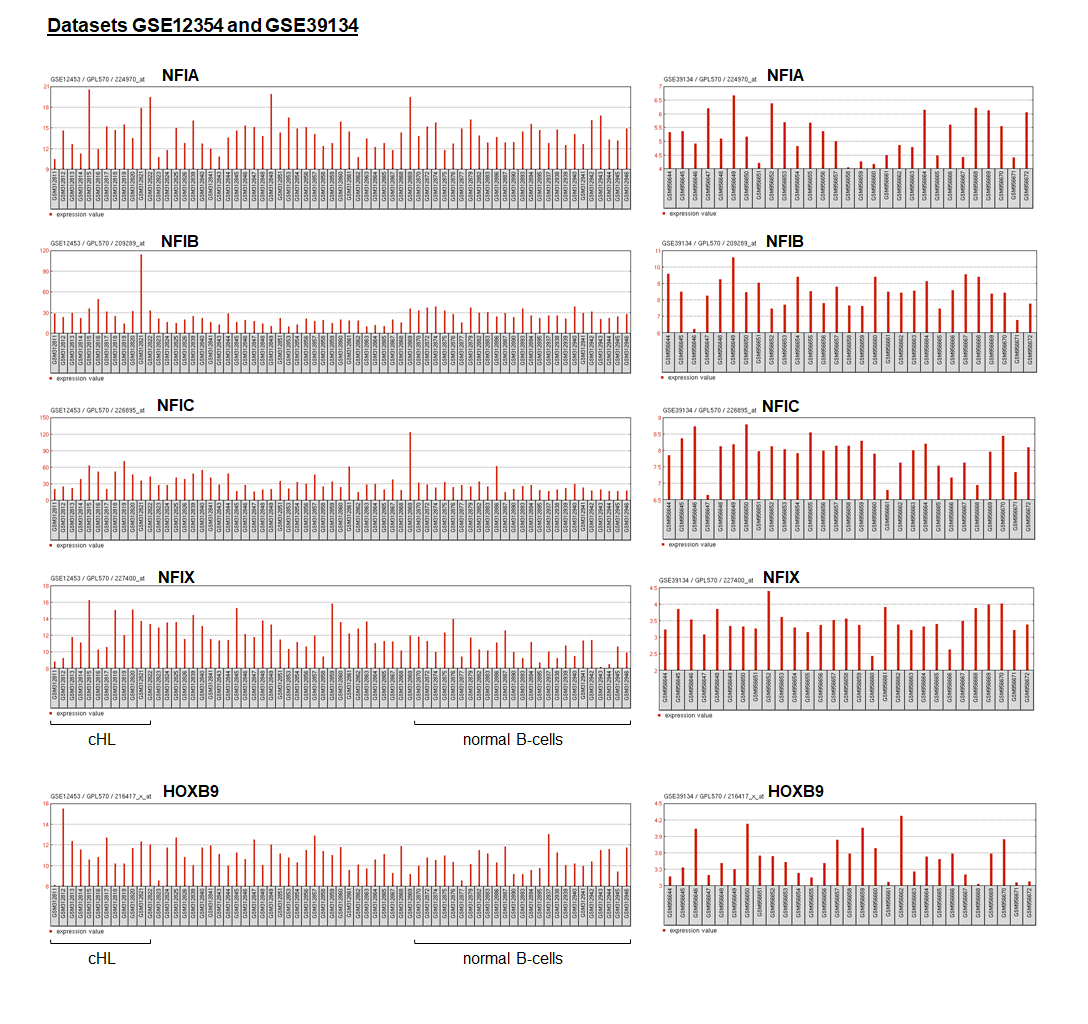

Supplement: S7 Fig — Gene expression analysis of NFI-family genes NFIA, NFIB, NFIC and NFIX in addition to HOXB9 using datasets GSE12453 and GSE39134. Samples of normal B-cells are indicated. (TIF) [file pone.0246603.s007.tif]

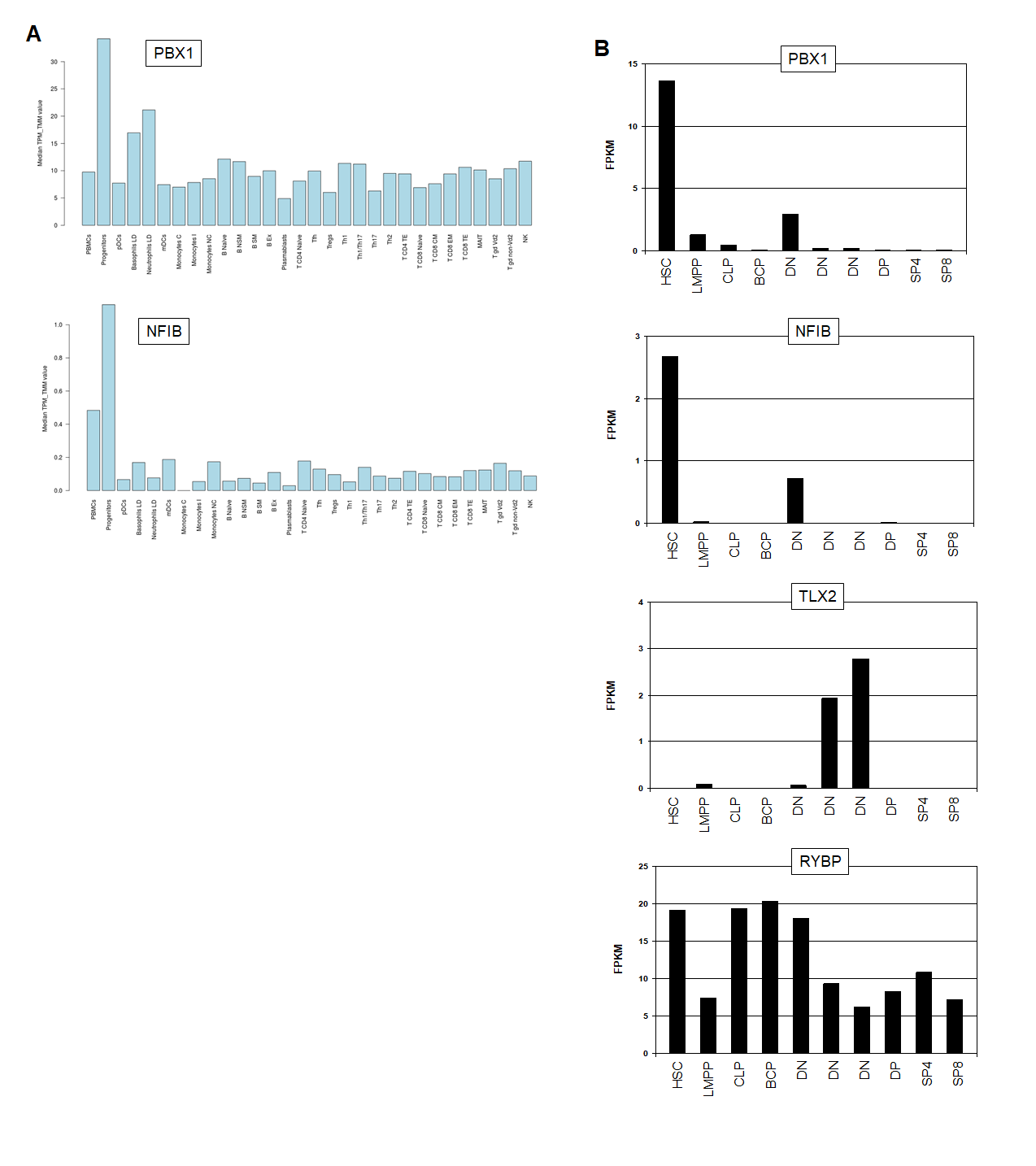

Supplement: S8 Fig — Gene expression analysis of four selected genes using datasets GSE107011 (left) and GSE69239 (right). (TIF) [file pone.0246603.s008.tif]

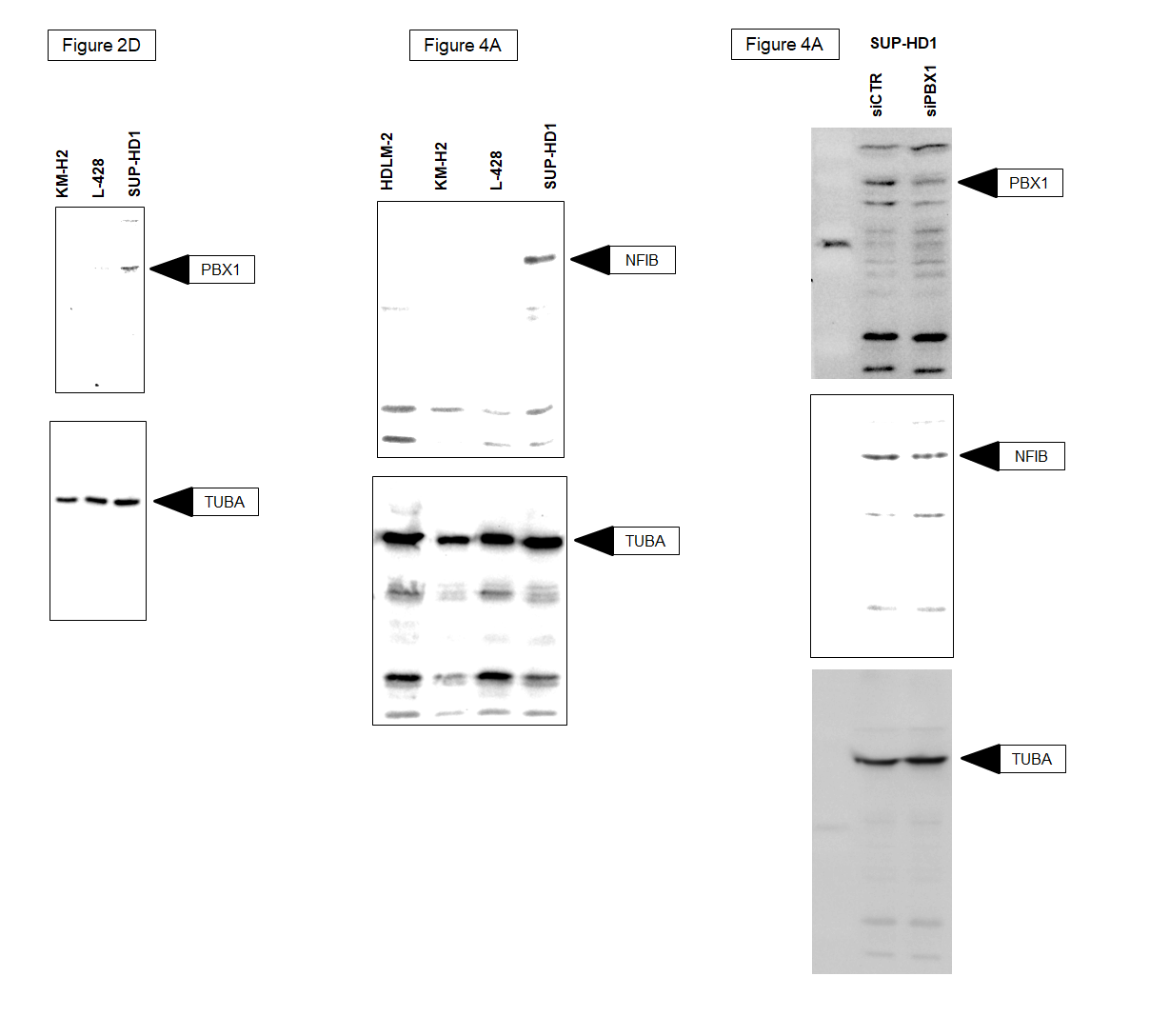

Supplement: S1 File — (TIF) [file pone.0246603.s012.tif]
